# The impact of social, national and community-based health insurance on health care utilization for mental, neurological and substance-use disorders in low- and middle-income countries: a systematic review

**DOI:** 10.1186/s13561-020-00268-x

**Published:** 2020-04-24

**Authors:** Sumaiyah Docrat, Donela Besada, Susan Cleary, Crick Lund

**Affiliations:** 1 Alan J. Flisher Centre for Public Mental Health, Department of Psychiatry and Mental Health, University of Cape Town, 46 Sawkins Road, Rondebosch, Cape Town, Western Cape 7700 South Africa; 2 Health Systems Research Unit, South Africa Medical Research Council, Cape Town, South Africa; 3 Health Economics Unit, University of Cape Town, Cape Town, South Africa; 4 Centre for Global Mental Health, Health Service and Population Research Department, Institute of Psychiatry, Psychology and Neuroscience, King’s College London, London, UK

**Keywords:** Social health insurance, National health insurance, Community-based health insurance, Mental health care, Developing countries, Health financing, Mental health, Health care utilization, Mental health care utilization, Mandatory health insurance

## Abstract

**Background:**

Whilst several systematic reviews conducted in Low- and Middle-Income Countries (LMICs) have revealed that coverage under social (SHI), national (NHI) and community-based (CBHI) health insurance has led to increased utilization of health care services, it remains unknown whether, and what aspects of, these shifts in financing result in improvements to mental health care utilization. The main aim of this review was to examine the impact of SHI, NHI and CBHI enrollment on mental health care utilization in LMICs.

**Methods:**

Systematic searches were performed in nine databases of peer-reviewed journal articles: Pubmed, Scopus, SciELO via Web of Science, Africa Wide, CINAHL, PsychInfo, Academic Search Premier, Health Source Nursing Academic and EconLit for studies published before October 2018. The quality of the studies was assessed using the Effective Public Health Practice Project quality assessment tool for quantitative studies. The systematic review was reported according to the PRISMA guidelines (PROSPERO;2018; CRD42018111576).

**Results:**

Eighteen studies were included in the review. Despite some heterogeneity across countries, the results demonstrated that enrollment in SHI, CBHI and NHI schemes increased utilization of mental health care. This was consistent for the length of inpatient admissions, number of hospitalizations, outpatient use of rehabilitation services, having ever received treatment for diagnosed schizophrenia and depression, compliance with drug therapies and the prescriptions of more favorable medications and therapies, when compared to the uninsured. The majority of included studies did not describe the insurance schemes and their organizational details at length, with limited discussion of the links between these features and the outcomes. Given the complexity of mental health service utilization in these diverse contexts, it was difficult to draw overall judgements on whether the impact of insurance enrollment was positive or negative for mental health care outcomes.

**Conclusions:**

Studies that explore the impact of SHI, NHI and CBHI enrollment on mental health care utilization are limited both in number and scope. Despite the fact that many LMICs have been hailed for financing reforms towards universal health coverage, evidence on the positive impact of the reforms on mental health care utilization is only available for a small sub-set of these countries.

## Introduction

In 2005, the World Health Assembly endorsed a resolution urging its member states to work towards sustainable health financing with a view to achieving universal health coverage (UHC) [1–3]. UHC is a system in which all individuals in a society are able to access the promotive, preventive, curative, rehabilitative and palliative health services they need without facing financial hardship, and that these services are of sufficient quality to be effective [1–4]. There is widespread recognition that the achievement of such a goal will rely on radical reforms in the existing health financing environments for most low- and middle-income countries (LMICs) if UHC is to be achieved according to the aforementioned dimensions [1, 2, 5–7]. In these contexts, increasing mandatory pre-payment funding is key to shifting away from high levels of out of pocket (OOP) payments for health care to protect individuals from the negative financial consequences of using health services and achieve equity in access [1–3].

Health financing reforms include changes in the way that revenues for health are generated and collected, how they are pooled to spread risks, the means by which the provision and purchasing of services are determined and how providers should be paid [5, 6]. While financing reforms towards mandatory prepayment have been repeatedly called for, there remains a lack of consensus about how LMICs should structure reforms aimed at moving towards UHC [8]. The WHO has advocated for social health insurance (SHI) and national health insurance (NHI) mandatory payment mechanisms as a priority to achieving equitable financing of health care and the achievement of UHC [1–3, 9].

Whilst in practice, the definition of these systems are often blurred; NHI is generally understood as a mandatory contribution scheme, with pooling of resources at the national level and a single purchaser model for purchasing a package of services for all citizens, regardless of whether they have contributed [9–11]. In this system, mandatory prepayment is comprised of general revenues of the government (generally a combination of taxes levied on individuals and firms; taxes levied on consumption, such as value added tax and customs duties, and; revenues from government owned enterprises particularly among countries where natural resources represent a substantial amount of government revenues) [12]. SHI includes mandatory contributions from certain groups; contributors may be all employed people, or defined groups in certain industries [12, 13]. Therefore, in a SHI model, universality can only be achieved if contributions are made on behalf of specific individuals in the population who are not able to afford contributions themselves. Thus, most countries that have adopted SHI reforms usually combine a number of different sources of funds, where government contributes on behalf of those that can’t afford to pay themselves. Although the focus of this study was initially conceived to examine mandatory health insurance systems (i.e. social or national health insurance); there has been evidence that voluntary community-based health insurance (CBHI) may play a role in systems transitioning toward UHC [14], particularly where there is a large population that falls outside of formal sector employment.

There has already been remarkable success among several LMICs in that these countries are considered to have *almost* achieved universal coverage through health financing reforms. Countries include Cambodia, Chile, Colombia, Costa Rica, Estonia, the Kyrgyz Republic, Philippines, Sri Lanka, Thailand, Tunisia, and Vietnam [7, 15]. Whilst no longer considered among the LMICs, China and South Korea have also been hailed as having health systems which have almost afforded UHC to their entire populations [7, 15]. As governments consider ways in which UHC goals can be achieved within their context, there have been appeals for greater sharing of knowledge such that meaningful lessons from the experiences of other countries in reforming health financing systems can be gathered, specifically with regards to their funding sources, pooling arrangements, purchasing methods and policies on benefits and patient cost-sharing [2, 9].

A key concern amongst many LMICs is the low priority afforded to mental health. Despite the prevailing successes in LMICs transitioning toward sustainable mandatory health financing systems, the burden of mental disorders is increasing globally, with 1.1 billion people affected by a mental or substance use disorder, worldwide causing 8% of all Disability-Adjusted-Life-Years (DALYs) and 18.5% of all Years Lived with Disability (YLD) [16, 17]. After cardiovascular disease and neoplasms, MNS disorders represent the third highest contributor to global DALYs [16] and the highest contributor to global YLD [16]. These statistics are echoed in low- and middle-income country (LMIC) settings with MNS disorders representing the 5th highest contributor to DALYs, accounting for the highest proportion of overall YLD [16].

A large multi-country survey supported by the WHO showed that 76–85% of people with severe mental disorders (psychosis, bipolar disorder and suicide attempt) in low-income countries had not received any treatment in the previous 12 months; whilst the treatment gap for minimally adequate treatment for major depression and anxiety exceeds 80% (83.5% and 90.2%, respectively) among LMICs [18–20]. There is concern that if mental health priorities are not explicitly defined and reflected in the financing policies and activities supporting the overall implementation of health financing reforms, true UHC inclusive of mental health care will not be achievable in LMIC contexts [21, 22]. A study on mental health financing challenges, opportunities and strategies for LMICs recently concluded that the inclusion of mental health in ongoing reforms to national insurance schemes represents one of the most promising avenues for sustainable mental health financing [21]. As emphasized by the Lancet Commission on Global Mental Health and Sustainable Development, achieving UHC must involve the explicit inclusion of mental health within reimbursement and mandatory insurance schemes as a standard, not as a complementary option [17]. Mental health and the treatment of mental, neurological and substance-use (MNS) disorders represent a good example of conditions which are afforded low policy priority and are frequently excluded from national and social health insurance schemes, especially in LMIC – despite the burden of disease for MNS disorders.

In light of the explicit inclusion of mental health within the SDG targets, recognizing its ongoing neglect health agenda, accumulating a body of evidence identifying the role of National Health Insurance mechanisms in increasing health care utilization, including for MNS disorders, will lend itself to the inclusion of mental health within the package of services being provided within countries aspiring towards UHC. Whilst several systematic reviews conducted in LMICs have revealed that coverage under social, national and community-based health insurance schemes has led to increased utilization of health care services, it remains unknown whether, and what aspects of, these shifts in financing result in improvements in mental health care utilization, thereby achieving the objectives of universalizing health care, inclusive of access to care for MNS disorders [14, 23, 24]. This study therefore aims to examine the impact of social, national and community-based health insurance on mental health care utilization in LMICs; and to identify whether there are any specific lessons that can be learnt from existing approaches to integrate mental health care into financing reforms towards universal health coverage. Further, the study aims to deriving meaningful lessons from innovative reform experiences of how countries have altered their funding sources, pooling arrangements, purchasing methods, and policies on benefits and patient cost-sharing to achieve better mental health care utilization [9].

## Methods

We developed a protocol for this review according to the PRISMA guidelines [25] and in 2018 we registered the protocol with PROSPERO, the International Prospective Register of Systematic Reviews (PROSPERO; 2018: CRD42018111576).

### Eligibility criteria

The inclusion and exclusion criteria are listed in Table 1. Studies were included if they: (i) adopted a quantitative research design or reported a quantification of mental health care utilization; (ii) examined the influence of national, social or community-based health insurance on mental healthcare utilization; (iii) were carried out in a low- or middle-income country either as per 1987 *or* 2017 definitions to allow for income changes over time; and (iv) were available in English. Studies were excluded if they were (i) qualitative descriptive studies, policy reviews, systematic reviews, opinion pieces, editorials, letters to the editor, book chapters, commentaries or conference abstracts; (ii) written in a non-English language, and; (iii) were conducted in a high-income country as at 1987 *and* 2017. Studies whose primary outcome was not mental health care utilization but provided a secondary analysis with comparisons of mental health care utilization by insurance status were also included. Studies that explored the impact of private health insurance on mental healthcare utilization were excluded, unless they were included as a comparison group. The present study defines MNS disorders as encompassing: Alcohol use disorders; Neurological disorders (Alzheimer’s disease and other dementias, Epilepsy); Illicit drug use disorders (Amphetamine use disorders, Cannabis use disorders, Cocaine use disorders, Opioid use disorders, Other drug use disorders); Eating disorders (Anorexia nervosa, Bulimia nervosa); Mood disorders (Anxiety disorders, Dysthymia, Major depressive disorder, Bipolar disorder); Psychotic disorders (Schizophrenia); Autism spectrum disorders (Autism, Asperger syndrome); Behavioural disorders (Attention-deficit/hyperactivity disorder, Conduct disorder); and Developmental disorders (Idiopathic developmental intellectual disability).

**Table 1:** Inclusion and exclusion criteria

Criteria	Inclusion Criteria	Exclusion Criteria
Study Design	Any quantitative study design	Qualitative studies unless they reported a quantification of mental health care utilization
Language	Available in the English Language	Unavailable in the English Language
Setting	Low- and Middle-Income counties either in 1987 or in 2017 to allow for income changes over time	High Income countries in 1987, that remained high income in 2017.
Publication	Peer-reviewed academic articles	Policy reviews, systematic reviews, opinion pieces, editorials, letters to the editor, book chapters, commentaries or conference abstracts
Topic	Studies the impact of community-based, national or social health insurance on mental healthcare utilization	Does not study the impact of community-based, national or social health insurance on mental healthcare utilization or, examines the impact of private health insurance on mental healthcare utilization.

### Information sources and search strategy

A systematic search for peer-reviewed articles published until October 1st, 2018 was conducted between 05 and 09 October 2018. We searched nine databases of peer-reviewed journal articles: Pubmed, Scopus, SciELO via Web of Science, Africa Wide, CINAHL, PsychInfo, Academic Search Premier, Health Source Nursing Academic and EconLit. The search strategy included the use of a combination of free text, indexing terms, database-specific limits (e.g. humans, English-language) and database-specific subject headings/vocabulary (e.g. Medical Subject Headings (MeSH)). Multiple search terms for each of the following three concepts were developed: (1) social, national and community-based health insurance; (2) mental health care, and; (3) low- and middle-income countries (Additional file 1). Within each concept, search terms were combined using the Boolean term ‘OR’. The three concepts were then combined using the Boolean term ‘AND’. Mental healthcare utilization was conceptualized as the use or consumption of any health services for the purpose of preventing, treating or obtaining information about one’s mental health problems or mental health status. Given the dearth of literature of this kind as well as varying opinions and definitions of utilization, we did not limit our search terms to publications that included the term “utilization”. The database search strategy for the systematic review and the full Pubmed search is provided as an additional file (see Additional file 1). Minor adjustments were made to adapt the strategy to the various electronic databases searched, for example, MeSH terms were removed when searches were conducted for all databases excluding Pubmed. Searches were limited to human studies. There were no publication date restrictions however only articles published or available in English were included.

### Screening and eligibility

Following the search of databases, the titles and abstracts of the search results were recorded and transferred into Endnote [26], where duplicates were identified and deleted. After irrelevant titles were excluded by one reviewer (SD), the titles and abstracts were double screened by SD and DB against the inclusion and exclusion criteria, tracking decisions using a pre-piloted form and dedicated table. Once the abstracts were screened, the full papers of the included abstracts, or of those for which more information was needed in order to include or exclude, were obtained and assessed for eligibility by both reviewers. Any full-text articles that could not be retrieved through the University of Cape Town Health Sciences Library electronic directory were sought via the inter-lending network in South Africa, or via electronic correspondence with authors. Differences between authors’ opinions were resolved via discussion throughout the review process. Agreement between the two reviewers was calculated by the kappa statistic.

### Data extraction and analysis

Data about and derived from the included papers were extracted by the first author (SD) onto a predesigned Excel-based data extraction form. The purposely designed, pre-piloted, spreadsheet of tables included: study source, design and participant characteristics, type and characteristics of the insurance mechanisms under examination including (where specified) revenue generation, pooling and purchasing arrangements as well as characteristics of the benefit package; the results for our primary outcome (mental health care utilization), other relevant secondary results, and the results of the study’s quality assessment. Quality of these studies was assessed by the Effective Public Health Practice Project (EPHPP) quality assessment tool for quantitative studies [27]. Quality criteria were not used in decisions regarding inclusion or exclusion of eligible studies. As the purpose of this review was to gain insight into the current state of the literature, including reporting styles, where data relating to our primary outcome (mental health utilization) was only presented in Figures, or no empirical data tables were included relating to our outcome of interest, corresponding authors were contacted once via electronic mail to obtain these data. No further efforts were made to contact authors for supplementary materials or clarifications outside of what was reported.

Given the heterogeneity of the study designs, the insurance mechanisms being examined, and the outcome measures of mental health care utilization reported in the identified studies, a meta-analysis was not conducted. Instead, a qualitative synthesis of findings is presented, which compares, evaluates and summarizes findings narratively in relation to the review questions. Descriptive statistics were calculated using STATA 13.

Before narratively synthesizing the impact of SHI, NHI or CBHI on mental health care utilization, given the lack of specific details regarding the particular financing mechanisms under examination in the included studies and to give context to the results, we have outlined the characteristics of the SHI, NHI and CBHI schemes that have been examined within the included papers, including their population coverage, revenue generation and pooling arrangements, benefits packages and provider payment mechanisms. These are provided as an Additional File based on a review of secondary sources (see Additional file 2).

## Results

### Search results and study selection

**Fig. 1:**
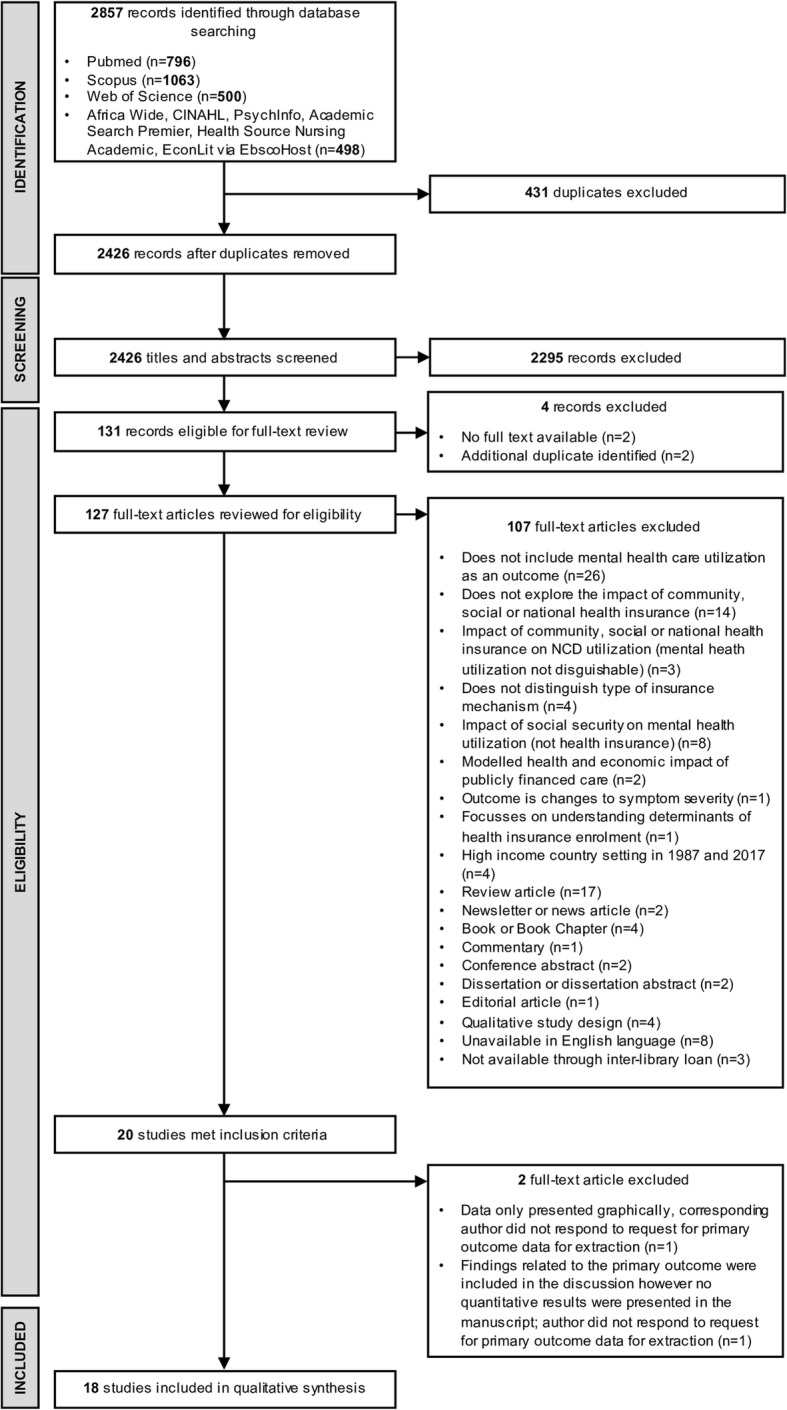
Results of database, abstract and full text screening

A total of 2857 articles were identified from databases, of which 2426 abstracts were screened for eligibility (Fig. 1). In total, 127 articles were selected for full-text review. Of these, 25% did not include any measure of mental health care utilization; 16% were review articles; and 13% did not explore the impact of community-based, social or national health insurance (i.e. focused on private health insurance only). English-language translations of articles written in other languages were not available for eight articles, whilst three articles could not be obtained from the inter-library lending facility of the University of Cape Town. Following the full-text review, we found twenty articles that met the inclusion criteria. Two full-texts were excluded at this stage as in one instance, our primary outcomes were presented graphically only (*n* = 1) and in the second, findings were mentioned in the discussion with no empirical data outlined in the results (*n* = 1); corresponding authors were contacted for access to these data and no responses were obtained. In total 18 studies were included in the final review. Reviewer agreement on selection of publications for final review was 94.2% (kappa = 0.81).

The majority of articles were published in Asia (*n* = 16, 89%), including twelve in China [28–39], two in South Korea [40, 41] and two in Thailand [42, 43]. One article was from Chile, South America [44] and one article reporting findings across 22 low-income, 17 lower-middle, and nine upper-middle countries as at 2003 [45] (Table 2). Thirteen of the 18 articles examined the impact of SHI on mental health care utilization [28–39, 44]; whilst three examined the impact of NHI [40, 41, 43] and one article examined the impact of CBHI [42] on mental health care utilization. The remaining paper explored a range of financing mechanisms across 48 LMICs [45]. Across the sample of studies, the approximate average duration between the establishment of the financing scheme and the data collection (i.e. period of examination) for the study was 10.9 years (range 1–24 years).

**Table 2:** Overview of the Included Studies

Author	Year	Continent	Country	Study Design	MNS Disorders	Period of Data Collection	Primary Health Insurance Scheme examined	Year of Scheme establishment	Approximate Duration between Establishment and Evaluation (years)
Asawavichienjinda, T., et al.	2003	Asia	Thailand	Cross-sectional study	Epilepsy	1997	CBHI	1983	14
Chung, W., et al.	2013	Asia	South Korea	Retrospective, cross-sectional study	Schizophrenia	2005 to 2006	NHI	1989	16
Hirunrassamee, S., et al.	2009	Asia	Thailand	Retrospective chart review	Epilepsy	2003 to 2005	NHI	2002	1
Hwang, J.E., et al.	2018	Asia	South Korea	Cross-sectional study	Depressive disorders and Anxiety Disorders	2013	NHI	1989	24
Araya, R., et al.	2006	South America	Chile	Cross-sectional study	Depressive disorders and Anxiety Disorders	1996 to 1998	SHI	1979	17
Ding, X., et al.	2018	Asia	China	Cross-sectional study	Epilepsy	2013 to 2014	SHI	1998, 2002, 2007	15
Feng, Y., et al.	2012	Asia	China	Retrospective Cross-sectional study	Schizophrenia	2010	SHI	1998, 2007	12
He, P., et al.	2017	Asia	China	Cohort study	Intellectual Disability	2007 to 2013	SHI	2002, 2007	5
Jian, W., et al.	2009	Asia	China	Difference in difference	Schizophrenia, Bipolar disorder, Vascular Dementia, Mental and behavioural disorder due to use of alcohol, Manic Episode or Depressive episode	2002 to 2006	SHI	1998, 2007	4
Wang, Z.-M., et al.	2015	Asia	China	Retrospective chart review	Schizophrenia-spectrum disorders; Bipolar disorder; Major depression	2007 to 2013	SHI	1998, 2002, 2007	9
Xu, J., et al.	2018	Asia	China	Retrospective chart review	Mental, Behavioral and Neurodevelopmental disorders (all F code diagnoses based on the ICD-10 code)	2005 to 2014	SHI	1998, 2002, 2007	7
Xue, Q., et al.	2014	Asia	China	Cross sectional study	Schizophrenia	2010	SHI	1998, 2007	12
Yu-tao, X., et al.	2007	Asia	China	Cross-sectional study	Schizophrenia	2005 to 2006	SHI	1998, 2007	7
Yu-Tao, X., et al.	2007	Asia	China	Cross-sectional study	Schizophrenia	2006	SHI	1998, 2007	8
Zhang, X.-Q., et al.	2015	Asia	China	Retrospective chart review	Mental, Behavioral and Neurodevelopmental disorders (all F code diagnoses based on the ICD-10 code)	2007 to 2013	SHI	1998, 2007	9
Zhou, Y., et al.	2017	Asia	China	Cohort study	Schizophrenia	2012 to 2014	SHI	1998, 2007	14
Zhou, Y., et al.	2014	Asia	China	Retrospective chart review	Mental, Behavioral and Neurodevelopmental disorders (all F code diagnoses based on the ICD-10 code)	2010 to 2013	SHI	1998, 2007	12
El-Sayed, A.M., et al.	2015	48 LMICs	22 low-income, 17 lower-middle, and 9 upper-middle countries (World Bank 2003)	Cross-sectional study	Depression and Schizophrenia	2002 to 2004	N/A	N/A	N/A

With respect to the MNS disorders for which utilization was examined, 33% (*n* = 6) of the articles included mental health care utilization for schizophrenia [29, 34–36, 39, 40]; 16.7% (*n* = 3) included mental health care utilization for epilepsy [28, 42, 43]; 11.1% (*n* = 2) included mental health care utilization for depressive and anxiety disorders [41, 44]; with one article examining utilization for intellectual disability [30]. Of the remaining articles, three examined mental health care utilization for all F-code diagnoses (mental, behavioral and neurodevelopmental disorders) based on the ICD-10 code [33, 37, 38]; whilst the remaining two articles focused on mental health care utilization for those living with schizophrenia, bipolar disorder, vascular dementia, mental and behavioural disorder due to use of alcohol, manic episode, depressive episode [31] and; schizophrenia-spectrum disorders; bipolar disorder and major depression [32], respectively.

### Study quality

The study’s quality ratings are reported in Table 3. In terms of quality, four studies were considered of strong methodological quality [30, 31, 33, 39], eleven were of moderate quality whilst the remaining three were considered of weak quality (Table 3). The primary reason for the majority of studies obtaining a moderate score was as a result of their cross-sectional study design, whilst those with a weak rating were scored low as a result of both a cross-sectional design and data collection based on the extraction of data from insurance claims databases.

**Table 3:** Methodological Quality of the Included Studies

Author	Year	Design	Selection bias	Confounders	Blinding	Data collection	Withdrawal and drop outs	Intervention integrity	Analysis	Score
Araya, R., et al.	2006	3	1	1	2	1	N/A	N/A	1	MODERATE
Asawavichienjinda, T., et al.	2003	3	1	1	1	3	N/A	N/A	1	WEAK
Chung, W., et al.	2013	3	1	3	1	2	N/A	N/A	1	WEAK
Ding, X., et al.	2018	3	1	1	1	1	N/A	N/A	1	MODERATE
El-Sayed, A.M., et al.	2015	3	2	1	1	1	N/A	N/A	1	MODERATE
Feng, Y., et al.	2012	3	1	3	1	2	N/A	N/A	2	WEAK
He, P., et al.	2017	2	1	1	1	1	1	N/A	1	STRONG
Hirunrassamee, S., et al.	2009	3	1	2	2	2	N/A	N/A	2	MODERATE
Hwang, J.E., et al.	2018	3	1	1	2	2	N/A	N/A	1	MODERATE
Jian, W., et al.	2009	2	2	1	2	2	N/A	N/A	1	STRONG
Wang, Z.-M., et al.	2015	3	2	2	2	1	N/A	N/A	2	MODERATE
Xu, J., et al.	2018	2	2	1	2	1	N/A	N/A	1	STRONG
Xue, Q., et al.	2014	3	1	1	2	1	N/A	N/A	1	MODERATE
Yu-tao, X., et al.	2007	3	1	1	2	1	N/A	N/A	1	MODERATE
Yu-Tao, X., et al.	2007	3	1	1	2	1	N/A	N/A	1	MODERATE
Zhang, X.-Q., et al.	2015	3	2	1	2	1	N/A	N/A	1	MODERATE
Zhou, Y., et al.	2017	2	2	1	3	1	N/A	N/A	1	STRONG
Zhou, Y., et al.	2014	3	2	1	2	1	N/A	N/A	1	MODERATE

Papers were assessed using the Effective Public Health Practice Project’s (EPHPP) Quality Assessment Tool for Quantitative studies [27]

1 = Strong; 2 = Moderate; 3 = Weak

### Characteristics of the included studies

The majority of studies included in this review adopted a cross-sectional design (*n* = 10) [28, 29, 34–36, 40–42, 44, 45]; with two cohort studies [30, 39], one difference-in-difference study [31] and five studies using a retrospective chart review design [32, 33, 37, 38, 43] (Table 4). The publication dates of the papers ranged between 2003 and 2018. Substantial variation can be observed between the sample sizes among the included studies (range: 72–132,316). Most studies made use of data that was not specifically collected with the intention of evaluating the impact of health insurance on mental health care utilization (i.e. data were extracted retrospectively from claims databases, patient charts, and electronic hospital records) [29, 31–34, 37, 38, 40, 41, 43]. As such, only five papers made use of prospective data collection [28, 35, 36, 39, 42]; and three made use of data from systematic surveys (i.e. Santiago Mental Disorders Survey, World Health Survey, National Sample Survey on Disability in China) [30, 44, 45].

**Table 4:** Methodology and Main Findings of the Included Studies

Author, Year	Location	Study design	Data Source	Type of Mental Health Care Examined	Target Population	Overall Sample size	Health Insurance Mechanism type		Health Insurance Mechanism name	Sample Size	Mental Health Care Utilization Outcome of Interest	Measure of Impact	Secondary Outcomes of Interest	Measure of Impact
Araya, R., et al., 2006 [44]	Santiago, Chile	Cross-sectional study	Santiago Mental Disorders Survey; Psychiatric symptoms were assessed with the Revised Clinical Interview Schedule (CIS-R); 1996–1998	Outpatient Care	Adults aged 16–64 years living in private households in Santiago	3824 (51% female)	Group of interest	SHI	**National Health Fund (FONASA)**	1439	Frequency of Mental Health Consultation within the previous six months	15.1%		
							Comparison group (1)	Private Health Insurance	**ISAPRES (Instituciones de Salud Previsional), Armed Forces and Teachers Union**	1905		29.2%		
							Comparison group (2)	Uninsured	**No health insurance**	480		18%		
Asawavichienjinda, T., et al., 2003 [42]	Pak Thong Chai district, Nakhon Ratchasima province, Thailand	Cross-sectional study	All data for adult (> 14 years) cases of epilepsy (two or more clinical afebrile seizures unrelated to acute metabolic derangements or to withdrawal from drugs or alcohol, or seizures occurring within a 24 h period) registered in the Registry of Epileptics who had visited a sub-district health care office or community hospital in 1997 in the district of Pak Thong Chai were extracted; interviews also conducted with patients and their caregivers.	Inpatient and Outpatient Care	Adult epileptics aged over 14 years living in Nakhon Ratchasima Province of Thailand	72 (60% female)	Group of interest	CBHI	**Health Card Scheme**	57	Compliance with antiepileptic drug (AED) regiments over the past year; on time, without fail, without manipulating dosage 100% of the time	88%		
							Comparison group (1)	Uninsured	**No health insurance**	15		68%		
Chung, W., et al., 2013 [40]	National, South Korea	Retrospective, cross-sectional study	Claims and service use data extracted from the repositories for all National Health Insurance and Aid claims	Inpatient Care	South Koreans who received inpatient care for schizophrenia between 2005 and 2006	58,287 (45% female)	Group of interest	NHI	**Korean national health insurance**	24,301	Proportion of Long Stay inpatients (> 6 months)	17%	Likelihood of Long Stay inpatients (> 6 months) in psychiatric hospitals	Base
							Comparison group (1)	Government subsidies for those who do not have economic capability, and cannot work	**Medical Care Aid 1**	30,241		61.06%; AID Type 1 beneficiaries were four times more likely than NHI beneficiaries to be long stay (OR 4.299, 95% CI: 4.024–4.593)		AID Type 1 beneficiaries showed an OR of 5.704 (95% CI: 4.877–6.671)
							Comparison group (2)	Government subsidies for those who do not have economic capability, and can work	**Medical Care Aid 2**	3745		48%		AID Type 2 beneficiaries an OR of 3.308 (95% CI: 2.713–4.034).
Ding, X., et al., 2018 [28]	Zhejiang, China	Cross-sectional study	Screening questionnaire was based on WHO screening questionnaires previously used in China and on the International Community-based Epilepsy Research Group (ICBERG) screening instrument followed by epilepsy specialists performing door-to-door investigations with a more specialized questionnaire in participants with suspected epilepsy from the first stage.	Inpatient and Outpatient care	Population of Zhejiang province	118 (58% female)	Group of interest	SHI	**Urban Employee Basic Medical Insurance (UE-BMI); Urban Residence Basic Medical Insurance (UR-BMI), or New Rural Cooperative Medical scheme (NRCM)**	98	Treatment gap for active epilepsy; proportion not receiving any antiepileptic treatment (traditional medicine or antiepileptic drugs) for active epilepsy among those with active epilepsy	52%		
							Comparison group (1)	Uninsured	**No health insurance**	20		90%		
El-Sayed, A.M., et al., 2015 [45]	22 low-income, 17 lower-middle, and 9 upper-middle countries (World Bank 2003)	Cross-sectional study	World Health Survey (WHS) 2002–2004	Inpatient and Outpatient Care	Populations of LMICs with diagnosed depression and schizophrenia	10,419 (Depression, *n* = 8762; Schizophrenia, *n* = 1657)	Group of interest	SHI or NHI	**Countries where most or all health services, including primary care, are provided by the government (even if private or NGO sector services may exist in parallel and some out-of-pocket expenses may exist).**	3797 Depression, *n* = 3437; Schizophrenia, *n* = 360	Receipt of treatment for depression or schizophrenia based on self-report	• Depression: 82.2% of those diagnosed with depression received treatment • Schizophrenia: 86.7% of those diagnosed with Schizophrenia received treatment	Attributable benefit defined as the degree to which insurance coverage mitigated treatment gaps relative to 100% for rural populations and for the poorest 50% of the sample	Among men, the attributable benefit of insurance among the poorest 50% was 53.1% for depression Among men, the attributable benefit of insurance among rural residents was 53.4% for depression,Among women, the attributable benefit of insurance among the poorest 50% was 24.7% for depression and 94.8% for schizophrenia.
							Comparison group (1)	Private health insurance	**Countries with no or minimal services provided by the government, or where only limited health services were provided by the government (e.g., for maternal and child health, HIV/ AIDS care, vaccinations, or for special groups such as children, elderly, impoverished).**	6622 Depression, *n* = 5325; Schizophrenia, *n* = 1297		• Depression: 37.1% of those diagnosed with depression received treatment • Schizophrenia: 53.3% of those diagnosed with Schizophrenia received treatment • In adjusted models among men, the uninsured had lower likelihood of treatment for depression (0.59, 95% CI 0.37–0.92). Among women, the uninsured were significantly less likely to receive treatment for schizophrenia (0.57, 95% CI 0.47–0.69); The poorest 50% were significantly less likely to receive treatment for depression (0.81, 95% CI 0.72–0.92)		
Feng, Y., et al., 2012 [29]	Changsa, China	Retrospective Cross-sectional study	Claims and service use data extracted from the repositories of the social insurance agencies, in addition to qualitative interviews and a field survey of policy documents and implementation methods	Inpatient Care	Population of Changsha, China, diagnosed with schizophrenia who made use of inpatient care in 2010	527	Group of interest	SHI	**Urban Employee Basic Medical Insurance (UE-BMI)**	70	Average Length of Inpatient Stay	50.6 days	Utilization of antipsychotics; prescription of FGA and SGA	Those with UE-BMI coverage were rarely prescribed FGA alone (3%) and most inpatients received SGA alone (58%). Inpatients covered by UR-BMI faced the opposite situation with most inpatients receiving FGA alone (42.5%) and the proportion receiving SGA alone (32.8%) was far less than UE-BMI inpatients.
							Comparison group (1)	SHI	**Urban Residence Basic Medical Insurance (UR-BMI)**	457		187.1 days		
He, P., et al., 2017 [30]	National, China	Cohort study	Second National Sample Survey on Disability follow-up investigations from 2007 to 2013; Children aged 0–6 years: Those who were suspected of having IDs were then tested in the developmental quotient (DQ) by the Gesell Developmental Inventory for a definite diagnosis with IDs (DQ < 76). Children aged 7–17 years were screened by interviewers using disability screening questionnaires at their homes. If the screening found that the subjects had an ID tendency, they would be referred to developmental paediatricians and psychiatrists to make the final diagnosis of IDs based on both intelligence quotient (IQ < 70) and adaptive behaviour.	Rehabilitative care (occupational, physical, and speech or communication therapy)	Children (0–10) and adolescents (11–17 years) living with intellectual disabilities across the 31 provinces of China	744 (41% female)	Group of interest	SHI	**Urban Residence Basic Medical Insurance (UR-BMI), or New Rural Cooperative Medical scheme (NRCM)**	222	Likelihood of Rehabilitation service utilization defined as likelihood of individuals receiving at least one rehabilitation service (occupational, physical, and speech or communication therapy) in the past 12 months	• With the exception of the first year of follow-up (2007); the remaining years showed a significantly lower likelihood of service use among the uninsured participants (2008–2013). • OR ranged from 0.50 in 2008 to 0.55 in 2013 (OR range 0.50–0.63)		
							Comparison group (1)	Uninsured	**No health insurance**	522				
Hirunrassamee, S., et al., 2009 [43]	Bangkok and two Provinces in the northeastern region, Thailand	Retrospective chart review	Hospital electronic diagnosis and drug dispensing databases were used as data sources. The records were available on an individual patient level. Data from the entire patient populations of the three hospital from three fiscal years—October 1, 2002, to September 30, 2005—were retrieved for this study.	Inpatient Care	Population of Thailand diagnosed as having epilepsy who visited or were admitted to any of the three hospitals under study between October 1, 2002, and September 30, 2005; and were treated with anti-epileptic drugs for no less than 90 consecutive days (to qualify as suffering epilepsy as a chronic condition rather than an occasional one)	439	Group of interest	NHI	**Universal Health Coverage scheme (previously 30 Baht Scheme)**	89	Utilization of new drugs (anti-epileptics which render better control of seizures with fewer side effects: lamotrigine 100 mg)	13%	Average drug cost (Baht) per seizure free case	7318.29 Baht among UHC beneficiaries; SSS 14,416.76 Baht; CSMBS 6623.55 Baht (the most cost-effective system for this disease condition)
							Comparison group (1)	Social Health Insurance	**Social Security Scheme (SSS)**	62		19%		
							Comparison group (2)	Social Health Insurance	**Civil Service Medical Benefits Scheme (CSMBS)**	288		31%		
Hwang, J.E., et al., 2018 [41]	National, South Korea	Cross-sectional study	Health Insurance Review and Assessment service (HIRA)-Aged Patient Sample database containing claim data on 1 million elderly patients, accounting for 20% of the elderly population in Korea. Data for Patients who were prescribed antidepressents in primary care settings between January and December 2013 were extracted.	Outpatient care	The elderly (> = 65) population in South Korea who were prescribed antidepressents in 2013	132,316 (67% female)	Group of interest	NHI	**Korean national health insurance**	119,106	Utilization of tricyclic antidepressants (TCAs) among elderly Koreans in primary care settings measured as the proportion of antidepressants prescribed that were TCAs	49.70%		
							Comparison group (1)	Government subsidies for those who do not have economic capability, and can/cannot work	**Medical Care Aid**	13,464		51.60%		
							Comparison group (2)	Government subsidies for Veterns	**Veterans Health**	178		54.5%; Patients with Veterans health coverage were 1.62 times more likely to be prescribed TCAs compared with those who had NHI		
Jian, W., et al., 2009 [31]	Beijing, China	Difference in difference	Data was extracted from the Hospital Information System (HIS).	Inpatient Care	Population of urban China hospitalized between 2002 and 2004 for Schizophrenia, Bipolar Affective Disorder, Vascular Dementia, Mental and behavioural disorders due to alcohol, Manic episodes or Depressive episode.	1137	Group of interest	SHI	**Urban Employee Basic Medical Insurance (UE-BMI); Urban Residence Basic Medical Insurance (UR-BMI)**	396	Length of Inpatient Admission	120.66 days		
							Comparison group (1)	GHI	**Government Health Insurance Scheme (GIS)**	212		98.89 days		
							Comparison group (2)	Uninsured	**No health insurance**	529		60 days		
Wang, Z.-M., et al., 2015 [32]	Beijing, China	Retrospective chart review	An extensive chart review was carried out, collecting data from an electronic chart management system (ECMS) for discharged patients aged 18 to 59 years.	Inpatient care	Patients receiving inpatient care at Beijing Anding Hospital (aged 18–59) with a primary psychiatric diagnosis (F-code)	19,982 (52% female)	Group of interest	SHI	**Urban Employee Basic Medical Insurance (UE-BMI); Urban Residence Basic Medical Insurance (UR-BMI), or New Rural Cooperative Medical scheme (NRCM)**	9865	Likelihood of Electroconvulsive therapy (ECT) use known for high risk of significant cognitive impairments	44%		
							Comparison group (1)	Uninsured	**Uninsured, either not registered with any of China’s health insurance schemes or living in a place of residence that is not their place of residence as registered**	10,117		56% ECT use was independently associated with less health insurance OR: 0.7		
Xu, J., et al., 2018 [33]	Shadong province, China	Retrospective chart review	Hospitals’ Electronic Health Records (EHR). The EHR data documents all inpatient expenses incurred during hospitalization in a detailed and itemized way.	Inpatient Care	Population of Shandong province with a primary psychiatric diagnosis (F-code)	9504(53% female)	Group of interest	SHI	**Urban Employee Basic Medical Insurance (UE-BMI); Urban Residence Basic Medical Insurance (UR-BMI), or New Rural Cooperative Medical scheme (NRCM)**	3215	Utilization rate measured by length of stay	70 days • UE-BMI: 137.52 days • UR-BMI: 63.70 days NCMS: 24.99 days	Utilization rate measured by frequency of hospitalizations	Frequency of hospitalization: 2 • UE-BMI: 3.96 • UR-BMI: 2.27 • NCMS: 1.91
							Comparison group (1)	Uninsured	**Uninsured, either not registered with any of China’s health insurance schemes or living in a place of residence that is not their place of residence as registered**	6289		45 days		Uninsured: 1
Xue, Q., et al., 2014 [34]	Wuhan and Wuxi cities, China	Cross sectional study	Claim records of inpatients with at least one schizophrenia- relevant diagnosis (ICD-10 code F20) in the year 2010 were derived from the two cities’ respective Urban Employees’ Basic Medical Insurance (UE-BMI) and the Urban Residents’ Basic Medical Insurance (UR-BMI) reimbursement databases in an anonymous form. G	Inpatient Care	Urban population of China with diagnosed schizophrenia (F20) receiving inpatient care and antipsychotic medication in 2010	2904 (45% female)	Group of interest	SHI	**Urban Employee Basic Medical Insurance (UE-BMI)**	2728	Coverage of second-generation antipsychotic medication excluding clozapine (SGA);	SGA: 53%;	Coverage of first-generation antipsychotics) FGA) and coverage of clozapine (CL)	FGA: 22% CL: 25%
							Comparison group (1)	SHI	**Urban Residence Basic Medical Insurance (UR-BMI)**	176		SGA: 53%;		FGA: 35% CL: 12%
Yu-tao, X., et al., 2007 [35]	Hong Kong and Beijing, China	Cross sectional study	Interviews with subjects in Hong Kong were randomly selected from patients diagnosed with schizophrenia attending the outpatient clinic of a university-affiliated general hospital; their Beijing counterparts, matched according to sex, age, age at onset, and length of illness, were recruited from patients with schizophrenia attending the Adult psychiatric Outpatient Clinic at Beijing Anding Hospital. Case notes were also reviewed.	Outpatient care	Clinically stable outpatients with schizophrenia in Beijing and Hong Kong between 2005 and 2006	505 (52% female)	Group of interest	SHI	**Urban Employee Basic Medical Insurance (UE-BMI); Urban Residence Basic Medical Insurance (UR-BMI), or Government Insurance Scheme (GIS)**	462	Treated with/prescribed Anticholinergic medication (ACM) known for a variety of side effects including the impairment of cognitive capacity	50%		
							Comparison group (1)	Uninsured	**Uninsured, either not registered with any of China’s health insurance schemes or living in a place of residence that is not their place of residence as registered**	43		33%		
Yu-Tao, X., et al., 2007 [35]	Hong Kong and Beijing, China	Cross sectional study	Clinically stable outpatients with schizophrenia were randomly selected and interviewed in Hong Kong (HK) and Beijing (BJ). Assessment instruments included the Structured Clinical Interview for DSM-IV, Brief Psychiatric Rating Scale, Simpson and Angus Scale of Extrapyramidal Symptoms, Barnes Akathisia Rating Scale and the Hong Kong and Mainland China World Health Organization Quality of Life Schedule-Brief version.	Outpatient care	Clinically stable outpatients with schizophrenia in Beijing and Hong Kong between 2005 and 2006	398 (49% female)	Group of interest	SHI	**Urban Employee Basic Medical Insurance (UE-BMI); Urban Residence Basic Medical Insurance (UR-BMI), or Government Insurance Scheme (GIS)**	359	Treated with/prescribed clozapine	13%		
							Comparison group (1)	Uninsured	**Uninsured, either not registered with any of China’s health insurance schemes or living in a place of residence that is not their place of residence as registered**	39		36%		
Zhang, X.-Q., et al., 2015 [37]	Beijing, China	Retrospective chart review	Extensive chart review was carried out, collecting data from an electronic chart management system (ECMS) for discharged patients aged 60 years and above	Inpatient Care	Geriatric (aged 60 years and older) inpatients with an F-code diagnosis treated between 2007 and 2013 in Beijing	2339 (59% female)	Group of interest	SHI	**Urban Employee Basic Medical Insurance (UE-BMI); Urban Residence Basic Medical Insurance (UR-BMI), or Government Insurance Scheme (GIS)**	1846	Proportion receiving Electroconvulsive therapy (ECT)	24.2%; Those with health insurance were significantly less likely to receive ECT, OR 0.6 (0.4–0.8)		
							Comparison group (1)	Uninsured	**Uninsured, either not registered with any of China’s health insurance schemes or living in a place of residence that is not their place of residence as registered**	493		46%		
Zhou, Y., et al., 2017 [39]	Guangzhou, China	Cohort study	Survey upon discharge from Guangzhou Huiai Hospital (Positive and Negative Syndrome Scale (PANSS), for clinical symptoms, Insight and Treatment Attitudes Questionnaire (ITAQ) for insight and treatment attitudes, drug attitude inventory (DAI) and family experience interview schedule (FEIS)) and follow up call one year later to determine medication use post-discharge	Inpatient Care	Patients aged 16–60 years who have diagnosed schizophrenia living in Guangzhou, China; and their caregivers	236 (46% female)	Group of interest	SHI	**Urban Employee Basic Medical Insurance (UE-BMI); Urban Residence Basic Medical Insurance (UR-BMI), or Government Insurance Scheme (GIS)**	105	Proportion discontinuing psychotropic medication one-year post-discharge	14%		
							Comparison group (1)	Uninsured	**Uninsured, either not registered with any of China’s health insurance schemes or living in a place of residence that is not their place of residence as registered (i.e also those registered with NRCM)**	131		35%		
Zhou, Y., et al., 2014 [38]	Guangdong province, China	Retrospective chart review	Hospitals’ Electronic Health Records (EHR) from Guangdong Psychiatric Hospital	Inpatient Care	Patients with any F-code diagnoses living in Guangdong, China who were discharged between 2010 and 2013	8478 (42% female)	Group of interest	SHI	**Urban Employee Basic Medical Insurance (UE-BMI); Urban Residence Basic Medical Insurance (UR-BMI)**	2055	Number of inpatient admissions	3.3	Likelihood of first, second or third hospitalization	GIS and BMI groups were 1.6 and 2 times more likely to be in a second hospitalization than others; 2.1 and 3 times more likely to be in a first hospitalisation, and; 5.3 and 4.8 times more likely to be in more than 3 hospitalizations
							Comparison group (1)	Government Insurance System (GIS)	**Government Insurance Scheme (GIS)**	276		4.1		
							Comparison group (2)	New Rural Cooperative Medical Scheme (NCMS)	**New Rural Cooperative Medical Scheme (NCMS) and*others***	4897		1.7		

### Measures and types of mental health care utilization examined

Four of the included studies explored the impact of health insurance on outpatient mental health care utilization, exclusively [35, 36, 41, 44]; with three publications exploring inpatient and outpatient mental health care utilization [28, 42, 45] and ten studies focusing on the impact of health insurance on inpatient mental health care utilization exclusively [29, 31–34, 37–40, 43]. Only one study explored the impact of health insurance on utilization of rehabilitative care (occupational, physical, and speech or communication therapy) [30].

Our primary outcome of interest, mental health care utilization, was operationalized in a number of different ways across the included studies. The length of inpatient admissions was used as a measure of utilization in four of the included studies; three of which defined length of stay in terms of duration of inpatient admissions [29, 31, 33] and one of which defined length of stay in terms of the proportion of patients who were deemed *long-stay* indicating that their inpatient admission lasted longer than 180 days [40]. Outpatient mental health consultations were used as an outcome in two of the included studies, one of which reported on the frequency of mental health consultations within the previous 6 months [44]; and the second reporting the likelihood of children aged 0–17 years with intellectual disability receiving at least one rehabilitation service (occupational, physical, and speech or communication therapy) in the past 12 months [30]. The absolute number of inpatient admissions over a 3 year period was used as a primary measure of mental health care utilization in one of the included studies [38]. Self-report of having ever received treatment for depression and schizophrenia among those diagnosed was used as a measure of utilization in one of the included studies [45].

Prescription of and compliance with medication was included as an outcome for eight studies included in this review. Compliance with medications was used as a measure of utilization in two of the included studies; one study reported on the rates of compliance with antiepileptic drug (AED) regimens over the past year defined as the number of patients that took their prescribed AEDs on time, without fail, without manipulating dosage, 100% of the time [42] whilst the second study reported on the proportion of patients that discontinued psychotropic medication for schizophrenia one-year post discharge from an inpatient facility [39]. In terms of prescription of medications, studies could be differentiated by whether they were comparing the prescription of any medication for an MNS disorder, and those that compared the prescription of more- or less-favorable medications for MNS disorders. Among studies that compared the prescription of any medication for MNS disorders, one examined the proportion of individuals with active epilepsy that did not receive any anti-epileptic medication (either traditional medicine or AEDs) [28].

Studies comparing more- or less-favorable medications for MNS disorders included one study which examined the utilization of *new* AEDs (lamotrigine 100 mg), which are considered to render better control of seizures with fewer side effects [43]. A second study examined the utilization of tricyclic antidepressants (TCAs) among elderly Koreans in primary care settings measured as the proportion of antidepressants prescribed that were TCAs [41]; here the authors note that whilst TCAs show superior efficacy, they cause a number of side effects and thus newer classes of antidepressants are preferred among the elderly. A third study operationalized mental health care utilization as the number of outpatients diagnosed with schizophrenia that were prescribed Anticholinergic Medication (ACM) known for a variety of side effects including the impairment of cognitive capacity [36] whilst a fourth study examined the influence of insurance status on the prescription of clozapine, described by the author to have potentially fatal side effects, despite its high efficacy, among clinically stable outpatients with schizophrenia [35]. A fifth study examined the impact of insurance enrollment on the prescription of first-generation, second-generation antipsychotic medications (excluding clozapine), and clozapine among those receiving inpatient care for schizophrenia [34].

Finally, two studies examined the impact of insurance enrollment on the likelihood of and proportion receiving electroconvulsive therapy (ECT) in inpatient settings where the authors note that ECT is known for a high risk of cognitive impairment [32, 37].

### Impact of SHI, NHI or CBHI on mental health care utilization

#### Length and frequency of inpatient admissions

Of the included studies examining the impact of insurance enrollment on the length or frequency of inpatient admissions [29, 31, 33, 38, 40]; four were conducted in China [29, 31, 33, 38] and one conducted in South Korea [40]. By and large, studies from China explored the impact of enrollment in SHI schemes: the Urban Employee Basic Medical Insurance (UE-BMI), Urban Residence Basic Medical Insurance (UR-BMI), New Rural Cooperative Medical scheme (NCMS) and the Government Insurance System (GIS) on length of inpatient admissions. UE-BMI targets formal sector workers on a mandatory basis; coverage for UE-BMI is 19% of the population. UR-BMI targets children, the elderly, the disabled, and other non-working urban residents but varies by region. Enrollment is voluntary for households; coverage for UR-BMI is 19·5% of the population. NCMS targets rural residents on a voluntary basis; coverage for NCMS is approximately 59·7% of the population, GIS targets only those working in the government sector. Jian et al. (2009) and Xu et al. (2018) both found that those enrolled in at least one of these health insurance schemes had a significantly longer length of inpatient admission when compared to the uninsured [31, 33].

The length of inpatient admission among the uninsured urban population of China who were hospitalized between 2002 and 2004 for schizophrenia, bipolar affective disorder, vascular dementia, mental and behavioural disorders due to alcohol, manic episodes or depressive episodes was 60 days, compared with 120·7 days and 98·9 days for those enrollled in UE-BMI or UR-BMI schemes and those enrolled in the GIS, respectively [31]. Similarly, the length of inpatient admissions among the uninsured population of Shadong province of China with a primary psychiatric diagnosis between 2005 and 2014 was 45 days, compared to those insured under UE-BMI, UR-BMI and NCMS with an average length of inpatient admission across all three schemes was 70 days (137·5, 63·7 and 25·0 days, respectively) [33]. As a secondary outcome, Xu et al. (2018) also reported that frequency of hospitalizations between 2005 to 2014 were higher among the insured groups, whereby those enrolled in UE-BMI, UR-BMI and NCMS had an average of 2 hospitalizations (4·0, 2·3 and 1·9, respectively) during this period, compared to the uninsured group who had an average of 1 hospitalization during this period [33].

Feng et al. (2012) examined the length of inpatient admissions among the population of Changsha, China diagnosed with schizophrenia who made use of inpatient care in 2010 [29]. For those enrolled in the UR-BMI scheme, the length of stay was significantly longer than for those enrolled in the UE-BMI scheme, 187·6 days compared to 50·6 days, respectively [29]. Nonetheless, the authors note that 81% of those enrolled in the UE-BMI scheme received treatment from a tertiary hospital compared with 73·3% of those enrolled in the UR-BMI scheme who received treatment from a secondary hospital [29]. Further, 58% of those enrolled in the UE-BMI scheme received more expensive second generation antipsychotics, compared with 33% of those enrolled in UR-BMI [29].

Among patients with any psychiatric diagnoses discharged from Guangdong Psychiatric Hospital between 2010 and 2013, those enrolled in the NCMS scheme had on average the fewest number of inpatient admissions (1·7) when compared to those enrolled in the UE-BMI or UR-BMI schemes (3·3 hospitalizations) and those enrolled in the GIS scheme (4.1 hospitalizations). As secondary outcomes, the authors report that those enrolled in the GIS, UE-BMI or UR-BMI schemes were 1·6 and 2 times more likely to be hospitalized a second time; 3·1 and 3 times more likely to have a third hospitalization, and; 5·3 and 4·8 times more likely have more than 3 hospitalizations, when compared to those enrolled in the NCMS scheme.

In South Korea, Chung et al. (2013) compared the proportion of patients who received inpatient care for schizophrenia between 2005 and 2006 that were hospitalized for longer than 180 days (i.e. were deemed *long-stay*) among those enrolled in the NHI scheme and those enrolled in either the Medical Care Aid 1 or Medical Care Aid 2 schemes [40]. The NHI scheme in Korea covers approximately 96% of the population; a 20% co-payment is required for inpatient care services included in the benefit package. Medical Care Aid schemes are those in which beneficiaries do not have the economic ability to make formal contributions to the NHI and their health care is subsidized fully (for Aid 1 beneficiaries) or for 85% of care (i.e. a 15% co-payment for Aid 2 beneficiaries) by government. The study found that among those enrolled in the NHI scheme, 17% were deemed long-stay, compared with 61% of those enrolled as Medical Care Aid 1 and 48% of those enrolled as Medical Care Aid 2 beneficiaries [40].

#### Outpatient mental health consultations

Of the included studies examining the impact of insurance enrollment on the outpatient mental health consultations; one was conducted in Santiago, Chile [44] whilst the second was conducted in China [30]. Araya et al. (2006) found that among those enrolled in the National Health Fund (FONASA), a social health insurance mechanism in Chile, 15·1% reported that they had received a mental health consultation within the previous 6 months, compared with 29·2% among those enrolled in private health insurance schemes and 18% among the uninsured population [44]. The authors note that despite higher prevalence of mental disorders and increased severity of disorders being exhibited among those with social health insurance coverage, compared to those with private health insurance coverage, their rate of consultation for these disorders were the lowest [44]. Health insurance exerted the strongest association with likelihood of consultations for mental disorders (OR = 2.72; 95% CI = 1·6, 4·6), favoring private health insurance enrollment [44].

A study by He et al. (2017) in China was the only identified study that examined the influence of insurance enrollment on the use of outpatient rehabilitation services (occupational, physical, and speech or communication therapy) [30]. The authors found that uninsured children aged 0–17 years with confirmed intellectual disability, with the exception of the first year of follow-up, showed a significantly lower likelihood of service use (i.e. between 2008 and 2013) over the past 12 months when compared with those enrolled in either the UR-BMI or NCMS schemes (OR ranged from 0.50 in 2008 to 0.55 in 2013) [30].

#### Ever having received mental health treatment

A study exploring the influence of insurance enrollment across 48 low- and middle income countries conducted by El-Sayed et al. (2015) examined the self-report of having ever received treatment for depression and schizophrenia among those diagnosed [45]. Among countries where most or all health services, including primary care, are provided by the government (defined as the *insured*, even if some private services and out-of-pocket expenses exist in parallel); 82.2% and 86.7% of those diagnosed with depression and schizophrenia, respectively, reported ever having received treatment [45]. Among countries where no or minimal services are provided by the government, or where only very limited services were provided (i.e. defined as the *uninsured*), 37.1% and 53.3% of those diagnosed with depression and schizophrenia, respectively, reported ever having received treatment [45].

When disaggregated by biological sex, in adjusted models among men, the uninsured had a lower likelihood of treatment for depression (0.59, 95% CI 0.37–0.92) whilst amongst women, adjusted models demonstrated that the uninsured were significantly less likely to receive treatment for schizophrenia (0.57, 95% CI 0.47–0.69) [45]. Further, the authors note that the poorest 50% of women were significantly less likely to receive treatment for depression (0.81, 95% CI 0.72–0.92) [45]. As a secondary outcome, El-Sayed et al. (2015) reported on the attributable benefit, defined as the degree to which insurance coverage mitigated treatment gaps relative to 100% for rural populations and for the poorest 50% of the sample [45]. The findings showed that among men, the attributable benefit of insurance coverage among the poorest 50% of the sample was 53.1% for depression [45]. Among women, the attributable benefit of insurance coverage among the poorest 50% of the sample was 24.7% for depression and 94.8% for schizophrenia. Among men, the attributable benefit of insurance among rural residents was 53.4% for depression [45].

#### Prescription of and compliance with medication

Asawavichienjinda et al. (2003) compared the rates of compliance with antiepileptic drug (AED) regiments over the past year among those enrolled in the Thai Health Card Scheme, a CBHI mechanism, versus the uninsured [42]. The study was based on data collected in 1997 in the Pak Thong Chai district of Thailand [42]. For those enrolled in the Health Card Scheme, 88% reported that their AEDs were taken as prescribed, on time, without fail, without manipulating the dosage, 100% of the time compared with 68% among the uninsured. The authors found that health insurance was significantly associated with compliance among their sample [42]. Similarly, a study conducted by Zhou et al. (2017) assessed the proportion of patients discharged from Guangzhou Huiai Hospital between 2012 and 2014 that discontinued use of psychotropic medications one-year post-discharge among those enrolled in the UE-BMI, UR-BMI or the government SHI schemes compared with the uninsured [39]. Among those insured, 14% reported discontinuing the use of medications 1 year post-discharge, compared to 35% among the uninsured [39]. The authors note that health insurance coverage was an independent predictor of compliance, reducing the financial burden of both medications and visits to prescribing physicians but also providing greater access to outpatient care and coverage for prescription drug costs [39].

With regards to the prescription of medications, Ding et al. (2018) examined the proportion of individuals with active epilepsy in Zhejiang, China, that did not receive any anti-epileptic medication (either traditional medicine or AEDs) between 2013 and 2014 [28]. The study compared those enrolled in the UE-BMI or UR-BMI SHI schemes with the uninsured; determining that 52% of the insured sample did not receive any anti-epileptic medication compared with 90% of the uninsured sample. In the same way, Yu-Tao et al. (2007) examined the influence of insurance status on the prescription of clozapine, described by the author to have potentially fatal side effects, despite its high efficacy, among clinically stable outpatients with schizophrenia in Hong Kong and Beijing throughout 2006 [35]. The study also compared those enrolled in the UE-BMI or UR-BMI SHI schemes with the uninsured; determining that 13% of the insured received clozapine, compared to 36% of the uninsured sample [35].

Another study comparing more- or less-favorable medications was conducted by Hirunrassamee et al. (2009) which explored the influence of enrollment in Thailand’s Universal Health Coverage scheme (a national health insurance mechanism), Social Security Scheme (SSS) and the Civil Service Medical Benefits Scheme (CSMBS) – the latter two mechanisms being considered social health insurance mechanisms [43]. Between 2002 and 2005, the utilization of *new* AEDs, which are considered to render better control of seizures with fewer side effects [43] was highest among those enrolled in the CSMBS scheme (31%) [43]. Among those enrolled in the NHI scheme, 13% received new AEDs whilst 19% of those enrolled in the SSS scheme received new AEDs [43]. As a secondary outcome, the authors explored the average drug cost per seizure free case, determining that those enrolled in the CSMBS scheme had the lowest cost per seizure free case (Baht 6624) compared with the UHC scheme (Baht 7318) and the SSS scheme (Baht 14,416) [43].

The utilization of tricyclic antidepressants (TCAs) among elderly Koreans in primary care settings by enrollment in Korea’s National Health Insurance scheme was examined by Hwang et al. (2018, 41). The authors compared the proportion of antidepressants prescribed that were TCAs among those enrolled in the NHI scheme, those enrolled as Medical Care Aid beneficiaries (i.e. lacking economic capacity to contribute to NHI) and those enrolled in the Veterans Health Insurance scheme [41]. As mentioned, whilst TCAs show superior efficacy, they cause a number of side effects and thus newer classes of antidepressants are preferred among the elderly [41]. Among those aged 65 years and older in 2013 enrolled in the NHI scheme that were prescribed antidepressants, 49.7% were prescribed TCAs compared to 51.6% among the Medical Care Aid beneficiaries and 54.5% among the Veterans Health beneficiaries [41]. Elderly patients enrolled in the Veterans health scheme were 1.6 times more likely to be prescribed TCAs when compared to those who were covered by NHI [41].

Yu-tao et al. (2007) examined the number of outpatients diagnosed with schizophrenia that were prescribed Anticholinergic Medication (ACM), known for a variety of side effects including the impairment of cognitive capacity, among those enrolled in either UE-BMI, UR-BMI or GHI with the uninsured in Hong Kong and Beijing between 2005 and 2006 [36]. Among those insured, ACMs were prescribed to 50% of the sample whilst among the uninsured, ACMs were prescribed to 33% of the sample [36]. Finally, a study conducted by Xue et al. (2014) examined the impact of insurance enrollment between UE-BMI and UR-BMI on the prescription of first-generation, second-generation antipsychotic medications (excluding clozapine) and clozapine among those receiving inpatient care for schizophrenia in 2010 in the cities of Wuhan and Wuxi, China [34]. Coverage of second-generation antipsychotics (excluding clozapine) were equivalent among those enrolled in the UE-BMI and UR-BMI schemes (53% of each respective sample) [34]. Coverage of first generation antipsychotics was higher among those enrolled in the UR-BMI scheme (35%) compared to the UE-BMI scheme (22%) whilst coverage of clozapine was higher among those enrolled in the UE-BMI scheme (25%) compared with those enrolled in the UR-BMI scheme (12%) [34].

#### Receipt of electroconvulsive therapy (ECT)

With regards to the receipt of specific inpatient therapies, both studies included examined the impact of insurance enrollment on the likelihood of and proportion receiving electroconvulsive therapy (ECT) in inpatient settings whereby, as mentioned, the authors note that ECT is known for a high risk of cognitive impairment, but associated with a shorter length of inpatient admission [32, 37]. Wang et al. (2015) found that among patients receiving inpatient care at Beijing Anding Hospital with a primary psychiatric diagnosis, 44% of those enrolled in the UE-BMI, UR-BMI or NCRM schemes received ECT compared to 56% of those uninsured [32]. ECT use was independently associated with less health insurance (OR: 0.7).

Similarly, Zhang et al. (2015) found that among geriatric (aged 60 years and above) inpatients with a primary psychiatric diagnosis, treated in Beijing between 2007 and 2013; 24.2% of those enrolled in either the UE-BMI, UR-BMI or GIS schemes received ECT compared to 46% among the uninsured group [37]. Those with health insurance were significantly less likely to receive ECT (OR: 0.6 (0.4–0.8)) [37].

To visualize differences in mental health utilization across the health insurance mechanisms, simple bar charts were prepared, and these are provided as an Additional File (see Additional file 3).

## Discussion

This systematic review reports on the impact of social, national and community-based health insurance enrollment on health care utilization for MNS disorders in low- and middle-income countries. The small number of included studies resulting from the original search strategy (18 articles from 2426 abstracts reviewed) speaks to the limited nature of the current evidence base. Overall, findings demonstrated that enrollment in SHI or NHI schemes increased utilization of mental health care. This was consistent for the length of inpatient admissions, the number of hospitalizations, outpatient use of rehabilitation services, having ever received treatment for diagnosed schizophrenia and depression, compliance with drug therapies and the prescription of more favorable medications and therapies when compared to the uninsured. However, following the approach of other systematic reviews which explored the impact of insurance enrollment on health care utilization, it was difficult to draw overall judgements on whether the impact of insurance enrollment was positive or negative for mental health care outcomes [7, 14, 15, 23, 24].

There were some notable exceptions to these overall trends. For example, in Chile, outpatient mental health consultations were less frequent among those enrolled in the SHI in comparison to the uninsured and those with private health insurance coverage, despite the prevalence of disorders and severity being higher among those enrolled in the SHI scheme. Since the study was conducted, Chile has embarked upon ambitious reforms towards universal health coverage, referred to as the Regime of Explicit Health Guarantees (AUGE), which provides an entitlement to a certain set of services for all members and was implemented in 2002–03. It remains unclear whether the AUGE reforms have impacted on these trends. Another notable exception was in South Korea, whereby NHI beneficiaries were less likely to be long-stay inpatients (i.e. admitted for longer than 180 days) when compared to Medical Care Aid beneficiaries, who are economically vulnerable and thus the majority, if not all, of their costs are covered by government subsidies. These findings may point to the influence of cost-sharing arrangements under NHI schemes – where a greater share of costs are carried by individuals (i.e. 20% for inpatient care under Korea’s NHI); utilization may be lower - however where a greater share of costs are subsidized and individuals do not make direct contributions to their cost of care, their utilization (or length of stays) were longer (i.e. for medical aid beneficiaries who were totally or partially exempt from cost-sharing). Across all studies, when utilization was defined as the length of inpatient admissions, studies that demonstrated longer length of inpatient admissions for those enrolled in insurance mechanisms did not unpack whether these lengths of stay were warranted based on the severity of the condition, or whether these long lengths of stay indicated some form of inefficiency in the health system. Similarly, without data regarding the severity of conditions, findings that indicate shorter lengths of stay do not directly point to a lack of insurance coverage (and therefore greater out of pocket spending) as a reason for these trends.

Thus, whilst the review has demonstrated that enrollment in CBHI, SHI or NHI schemes increases utilization of mental health care compared to uninsured populations; the clinical complexity of mental health care, particularly for severe mental health disorders, compounded by cultural norms around medication use, was also revealed in this review. Other complexities included the implications of a lack of explicit treatment guidelines and mechanisms by which perverse provider incentives can be mitigated. Across studies from China, insurance coverage was associated with the increased prescription of ACM and anti-epileptic medication but the reduced provision of Clozapine and ECT for Schizophrenia; although importantly, prescription for all these drugs across all insurance groups was higher than globally recommended [28, 32, 35, 37, 39, 42, 43]. Each of these drugs is associated with negative side effects including the impairment of cognitive capacity, while clozapine is principally recommended for use with treatment resistant schizophrenia and is associated with increased cardiovascular risk and psychotic relapse and the potential for agranulocytosis [46]. There appears to be an improved alignment to global treatment recommendations amongst the insured, with the overprovision of unnecessary services to those paying out-of-pocket. Similarly, it was noted that ECT is associated with shorter length of inpatient stay, and therefore uninsured patients and/or their providers may be opting for it in greater frequency for earlier discharge however, ECT is recommended only for treatment resistant depression, and the rates of ECT in China in both arms (particularly the uninsured) seemed to be very high. The determination of adequate and appropriate care cannot be made without the assessment of patient outcomes; these have not been presented in any of the studies included.

In addition to the comparison of SHI, NHI, CBHI and uninsured healthcare, reimbursement mechanisms appear to play a critical role. Providers are reimbursed on a Fee for Service (FFS) basis in China, known to create a perverse incentive for excessive or expensive treatments. While FFS still prevails, province- and city-based reforms in China have explored capitation, pay-for-performance (PFP), and diagnosis-related groups (DRGs) for inpatient care [31, 47]. Switching to PFP approaches alone or in combination with capitation was found to reduce spending on drugs but has not had an impact on drug prescriptions in larger contexts [48]. The remainder of insurance mechanisms included in the review reimburse providers through a per capita or capitation-based mechanism, while a few also report a DRG payment mechanism adjusted for risk. Several studies note that private insurance members receive access to more expensive drugs than those enrolled in social health schemes, and authors call for reimbursement reforms as a key strategy within health insurance reform.

While the evidence may suggest that the uninsured are provided with unnecessary treatments in order to reduce their length of admission, the longer inpatient stays and increased numbers of hospitalizations amongst the insured may not necessarily be indicative of improved service quality or appropriate access. The large focus of the papers on inpatient mental health care utilization and severe mental health conditions may speak to a lack of developed outpatient and community based services; it may also point to the explicit inclusion of inpatient care as part of the benefits package without the explicit inclusion of outpatient services including rehabilitation. Further, as revealed through our secondary review of the insurance mechanisms under evaluation in this study, differences in cost-sharing arrangements for inpatient and outpatient care may lead more mental health care users to opt for inpatient care where a larger proportion of costs are covered, compared to outpatient care where (if available) co-payments are far higher. In the case of South Korea, for example, NHI enrollees face a 20% co-payment for inpatient care whilst they face co-payments ranging from 30 to 60% for outpatient care depending on the level of provider. Prior to reforms in Chile, FONASA enrollees faced up to 15% co-payments for inpatient care provided by the government, with low income groups facing no co-payments – however these enrollees faced important exclusions in terms of the services they were able to access. These findings suggest that in countries pursuing SHI and NHI, inpatient benefits may be more explicitly defined, when compared to entitlements for beneficiaries for outpatient care which may be covered under an umbrella of a primary health care package of services; thus subject to more provider rationing within capitation systems.

While countries implementing SHI mechanisms have made considerable progress in improving population health access, social assistance to cover vulnerable populations and cross-subsidization has been integral to achieving UHC [49]. Countries explored in this review including China, South Korea, Thailand and Chile all reported pro-poor policies consisting of staggered copayments relating to income, the exclusion of poor-income households from mandatory contribution to the NHI or the creation of specific insurance schemes subsidized by government for low income and vulnerable populations. However many of the intermediate mutual health insurance and community-based schemes that were created have been hampered by adverse selection, poor regulation and inadequate administrative capacity [50]. Furthermore, while such insurance schemes for poor income households have contributed to increased health access, the typically smaller benefit package, geographical fragmentation of services and reduced risk pooling associated with these schemes challenge the attainment of equity in service provision, and therefore Universal Health Coverage. For example, in China, mental health care utilization was routinely lower amongst those enrolled in the NCMS scheme which targets rural residents on a voluntary basis, and the UR-BMI scheme which targets children, the elderly, disabled and other non-working urban residents, when compared to the UE-BMI scheme for formal, salaried workers. Although SHI schemes hold potential to improve financial protection and improve utilization, in contexts such as China which began its transition to UHC by initially covering those with regular, salaried employment, these schemes have not evolved to include the rest of the population – and the emergence of additional schemes such as the NCMS and UR-BMI suggest that there are inequalities in the entitlements of beneficiaries across schemes, within countries [10]. As a result, improvements in coverage and utilization gained through multiple SHI schemes largely manifest as more individuals belonging to an explicit insurance scheme, rather than greater financial access and utilization of comprehensive services across the entire population [10].

In Thailand, hailed to have achieved UHC, largely based on general taxation, disparities exist across its three different insurance schemes, which include the civil servants’ medical benefit and social security scheme and the universal coverage scheme that includes 72% of the population [51]. The study from Thailand demonstrated that the utilization of *new* AEDs was highest among those enrolled in the CSMBS scheme (31%), with 19% and 13% of those enrolled in the SSS and NHI schemes respectively received new AEDs. Similarly, while 95% of China’s citizens are reported to have basic insurance under one of their three major schemes, only the UE-BMI requires mandatory contributions whilst government subsidies account for 75–85% of the premiums of the other two [52]. The lack of integration of the insurance programmes, both across regions and policies, has resulted in a fragmented risk pool and inequities in health access, particularly for rural populations who are not covered for service use in urban cities where the large psychiatric hospitals are located. South Korea has a combination of a National Health Insurance mechanism in place for 97% of the population funded through income tax and the remainder of the population within one of two public assistance programmes for low-income families. The reviewed study found that a larger proportion of the population belonging to the Medical Aid schemes were deemed long-stays. Medical Aid beneficiaries in Korea have been found to have poor health status and to receive insufficient healthcare services with the life expectancy difference between National Health Insurance beneficiaries and Medical Aid beneficiaries found to be 15.8 years for men and 8.9 years for women in 2017 [52]. Prior to reform, Chile also implemented multiple insurance mechanisms with 75% of the population belonging to the public scheme and 18% belonging to private insurance. The system reflects segmentation, inefficiencies and inequalities, with the most vulnerable groups being largely affected [53]; this study showed that two times as many people belonging to the private insurance scheme receiving mental health consultations, with NHI beneficiaries receiving even less than the uninsured population.

Given the highly stigmatized nature of mental health, amplified by the socioeconomic vulnerabilities experienced by many of those living with mental health disorders, explicitly outlining the inclusion of mental health within national and social health insurance benefit packages is key. Countries such as China and Thailand have made explicit inclusion of mental health during financial reforms including China’s 686 Programme in 2004, named after the initial funding allocation of 6.86 million yuan (equivalent to US$1 million today) [54]. Thailand produced its 2008 Mental Health Act resulting in mental health costs being absorbed under the country’s universal health coverage scheme and requiring officials to monitor and measure implementation [55], while Chile has now mandated that 56 priority diseases [56], including mental health, be offered across both insurance schemes. Defining the benefits package for mental health is however dependent on the availability of strong evidence related to the costs and benefits of treatments, heterogeneity of patient needs and preferences, the financing mechanisms being considered and the availability of infrastructure and services [57].

Despite some guidance by key actors on how countries can improve the design and functioning of their health systems to achieve UHC; countries on their path towards universal coverage are grappling with policy re-definition as well as cost containment, quality of care, equity, and regulatory considerations. Thus, the achievement of UHC, or at least progress towards it, still has not led to a major conversion between different countries when it comes to access and quality of service delivery. A review of lessons from 11 countries implementing UHC highlights its complexity [49]. The study demonstrated that moving towards UHC required long-term policy engagement inclusive of both technical knowledge and political commitment to invest in the development of solid institutional foundations, administrative capacity, and good governance to design and implement coverage-enhancing reforms that are inclusive and sustainable in the long run. The paper concludes that countries have a better chance of progressing in their efforts to design and implement coverage enhancing reforms that are inclusive, sustainable and of adequate quality if leaders demonstrate political commitment to reform, a clear understanding of the political economy challenges, and a willingness to learn from experience and adapt [49]. Evaluating the impact of UHC schemes is a methodologically challenging endeavor requiring both econometric skills to account for issues such as potential selection bias or the bidirectional relation that exists between effects of a UHC scheme and health status, in addition to the quality of information to adequately measure outcomes of interest and correct for the possible differences among intervention and comparison groups. Furthermore, it is critical to have sufficient knowledge of the specific scheme being evaluated including information around additional factors that may impact the outcomes being evaluated.

The assessment of UHC within countries implementing NHI or SHI mechanisms is challenging given that the available evidence rarely explores the causal links between the design features of the schemes and the outcomes observed [15]. In practice, this is indeed difficult to demonstrate given that there exist many confounders related to poorer populations having worse mental health, worse physical health, higher comorbidities, lower levels of education – all of which may serve as barriers to accessing health insurance entitlements. UHC monitoring challenges include sourcing reliable data on health service coverage and financial protection, being able to disaggregate it to expose coverage inequities and being able to measure effective coverage as it relates to the quality of service provision and its impact on mental health [58].

Health-related outcomes (such as access, health status, and financial protection) are affected by many more variables than UHC alone. UHC schemes may improve access by making services more affordable but they may not influence.

other dimensions of access such as limited availability of services or limited acceptability of existing health services.

UHC in itself does not have a direct impact on health but rather impacts on individual or household utilization of health services by reducing the financial barriers to access. Impact evaluations should therefore concentrate on measures of health status that can be attributed to this increased access to health. Furthermore, the impact of UHC schemes may vary across different populations, in which the impact of reducing direct payments on utilization might be stronger for poorer populations. Similarly, the impact of UHC schemes on nonmedical consumption, which represents a proxy for financial protection, may also be stronger among poorer households. Most research focuses on horizontal equity with regards to using equity of utilisation as a proxy indicator for equity of access. As such, research focuses on services and interventions for which there is readily available data at the expense of adequately defining or measuring unmet need for the most marginalized and disadvantaged populations [59], this is particularly relevant for mental health services.

Frenz et al. (2019) have developed an analytical framework for assessing equity of access in UHC policies by reflecting on both demand and supply side factors [59]. With the framework*, “equitable access is seen as the experiences and interactions of different socioeconomic groups with the health care system, within the broader context of the structural inequities that define social hierarchies and hence determine differential health needs*” (Rodney AM et al. 2014).

Furthermore, UHC schemes are not homogeneous interventions, even within countries, and despite them having a common goal, implementation varies. Complimentary schemes co-exist which vary with regards to the degree of coverage of services, levels of copayments, conditions of access and time of implementation-all likely to impact health outcomes. In addition, the lack of access to a UHC scheme cannot be assumed to be equivalent to the total lack of coverage in all settings, as UHC schemes may supplement or overlap with already existing publicly financed health systems. It is therefore important to carefully take into account this overlap when designing impact studies and interpreting their results [15].

The majority of papers included in the review did not describe the insurance schemes and their organizational details at length, nor did they define the explicit mental health entitlements for mental health care users, with limited discussion of the potential links between these features and the outcomes. As such, inferences were made by the authors of this paper based on the additional literature review. Despite the difficulty in establishing the impact of the individual financing functions of the insurance schemes on mental health utilization there is an association between increased mental health service utilization and increased insurance enrollment, likely largely because of the removal of financial barriers to access.

To the best of our knowledge, this systematic review is the first of its kind to systematically explore effects of health insurance schemes on mental health service utilization in LMICs. Further, it has facilitated a collection of supplementary data on health insurance schemes in countries of included studies. Despite some heterogeneity between countries, this review has demonstrated that the pursuit of NHI and SHI as a means of achieving UHC has the capacity to improve service utilization for MNS disorders.

This review has several limitations that are worthy of note. Firstly, only studies available in English were included. Secondly, the study did not examine whether improvements in utilization were pro-poor, nor did we take into account the mental health outcomes or severity of illness that may impact utilization as this information was not provided by the majority of included studies. Furthermore, given the cross-sectional nature of the majority of studies, only associations between insurance enrollment and mental health care utilization could be made, and causality could not be proved. In addition, studies that depended on self-reporting stood the risk of recall and social desirability bias. Future research must include a wider documentation of the impact of mental health care utilization among all countries adopting reforms toward UHC, with an explicit focus on how countries have altered their funding sources, pooling arrangements, purchasing methods, and policies on benefits and patient cost-sharing to achieve better mental health care utilization.

## Conclusion

Despite the fact that many LMICs have been hailed for financing reforms towards universal health coverage, it is surprising that evidence on the impact of the reforms on mental health care utilization is only available for a small sub-set of these countries, namely Thailand, China, South Korea and Chile. In addition there was very limited examination of the impact of enrollment on outpatient and community-based mental health care; LMICs transitioning to financing systems that pursue the goal of universalizing health care inclusive of mental health care do not yet have sufficient evidence to guide decision making on how to make the best use of available resources in order to achieve UHC, including considerations of the redistribution of resources from hospi-centric care to the community; task-shifting mental health care to non-specialist providers who receive ongoing specialist supervision; the initiation of early interventions that are accessible to at risk populations; integration of mental health in broader primary health care [17, 60–67]. Given that the social determinants of health influence the equity of coverage, the WHO recommends that UHC is assessed via a spectrum ranging from inputs and processes, to outputs, outcomes and impact [68]. Rather than measuring coverage, countries should focus on a subset of services and indicators that reflect overall quantity, quality, equity and financing of services, disaggregated by key socio-economic variables such as income, occupation, or disability. It is therefore important to also explore the impact of equitable UHC on access to a basic package of essential services and health outcomes for the entire population, most commonly disaggregated by geographical area, socio-economic status and gender [69]. Further, defining explicit benefit packages within national and social health insurance schemes is recommended, particularly for outpatient care, given the potential for: empowerment of poor and marginalized groups through explicit entitlements; improvements in efficiency and affordability; reductions in the risk of informal payments, and; guarantees of minimally adequate treatment irrespective of scheme enrollment.

## Supplementary information

**Additional file 1.**

**Additional file 2.**

**Additional file 3.**

## Data Availability

All data generated or analysed during this study are included in this published article [and its supplementary information files].

## Abbreviations

ACM: Anticholinergic Medication
AED: Antiepileptic Drug
AUGE: Regime of Explicit Health Guarantees
CBHI: Community Based Health Insurance
CSMBS: Civil Service Medical Benefits Scheme
ECT: Electroconvulsive therapy
EPHPP: Effective Public Health Practice Project’s Quality Assessment Tool for Quantitative studies
FONASA: National Health Fund
GIS: Government Insurance System
LMIC: Low- and Middle-Income Country
MNS: Mental, Neurological and Substance-Use
NCMS: New Rural Cooperative Medical scheme
NHI: National Health Insurance
OOP: Out-of-Pocket
SHI: Social Health Insurance
SSS: Social Security Scheme
UE-BMI: Urban Employee Basic Medical Insurance
UHC: Universal Health Coverage
UR-BMI: Urban Residence Basic Medical Insurance
