# Supplementary material for: The impact of social, national and community-based health insurance on health care utilization for mental, neurological and substance-use disorders in low- and middle-income countries: a systematic review
Source: Health Econ Rev. 2020 Apr 24;10:11. doi: 10.1186/s13561-020-00268-x (PMC7181535; doi:10.1186/s13561-020-00268-x)
Supplement: Supplementary file 1 — Additional file 1. [file 13561_2020_268_MOESM1_ESM.pdf]

PubMed Search:

Overview of Search Strategy

Health Insurance	AND	Mental Health Care Utilization	AND	LAMIC	NOT	Limits
National Health Insurance OR Social Health Insurance OR Community-based Health Insurance		Mental Health Services OR Mental Health Care		Low- and Middle-Income Countries (1987 – 2017)		High Income Countries (1987 and 2017)

Health Insurance Terms
National Health Insurance OR Social Health Insurance OR Community-based Health Insurance OR Community based Health Insurance OR Community Health Insurance OR Mandatory Health Insurance OR Informal Health Insurance OR Single-payer system OR Single Payer System
"National Health Programs/economics"[Mesh] OR "Insurance"[Mesh] OR "Health Care Sector/economics"[Mesh] OR "Health Care Sector/organization and administration"[Mesh] OR "Financing, Organized"[Mesh] OR "Healthcare Financing"[Mesh] OR "Health Equity/economics"[Mesh] OR "Universal Coverage/economics"[Mesh] OR "Health Care Reform/economics"[Mesh] OR "National Health Programs/legislation and jurisprudence"[Mesh] OR "National Health Programs/organization and administration"[Mesh] OR "Universal Coverage/legislation and jurisprudence"[Mesh]

Mental Healthcare Terms
Mental Health Services OR Mental Health Service OR Mental Healthcare OR Mental Health Care OR Mental Health System OR Psychiatric Services OR Psychiatric Care OR Psychiatry Services OR Psychiatric Health Care OR Psychiatric Healthcare OR Psychiatry OR Mental Illness OR Mental Health OR Severe Mental Disorder OR Severe Mental Disorders OR Common Mental Disorder OR Common Mental Disorders OR Mental Illness OR Mentally Ill Persons OR Substance Disorder OR Substance Disorders OR Substance Abuse OR Substance-Use Disorder OR Substance Use Disorder OR Substance-Use Disorders OR Substance Use Disorders OR Alcohol Use Disorder OR Alcohol Use Disorders OR Alcohol Abuse OR Alcohol-Related Disorder OR Alcohol Related Disorder OR Alcohol Addiction OR Opioid Abuse OR Opiate Addiction OR Opioid-Related Disorder OR Opioid Related Disorder OR Cannabis-Related Disorder OR Cannabis Related Disorder OR Cocaine Related Disorder OR Cocaine-Related Disorder OR Cocaine Addiction OR Amphetamine-Related Disorder OR Amphetamine Related Disorder OR Amphetamine Addiction OR Heroin Dependence OR Heroin Abuse OR Heroin Addiction OR Substance Induced Psychoses OR Substance-Induced Psychoses OR Anxiety Disorders OR Anxiety Disorder OR Bipolar Disorder OR Bipolar Disorders OR Manic-Depressive Psychosis OR Manic Depressive Psychosis OR Bipolar Affective Psychosis OR Bipolar Affective Psychosis OR Manic-Depressive Psychoses OR Manic Depressive Psychoses OR Bipolar Affective Psychoses OR Bipolar Affective Psychoses OR Bipolar Depression OR Anorexia Nervosa OR Binge-Eating Disorder OR Bulimia Nervosa OR Anorexia OR Binge Eating OR Binge-Eating OR Bulimia OR Depressive Disorders OR Depressive Disorder OR Depression OR Unipolar Depression OR Unipolar Depressions OR Mood Disorder OR Mood Disorders OR Dementia OR Dementias OR Attention Deficit Disorders OR Attention Deficit Disorders OR Conduct Disorder OR Conduct Disorders OR Neurocognitive Disorder OR Neurotic Disorder OR Neurodevelopmental Disorder OR Developmental Disability OR Development Disability OR Developmental Disorder OR Development Disorder OR Autism Spectrum Disorder OR Asperger Syndrome OR Autistic Disorder OR Autistic Disorder OR Autism Spectrum Disorders OR Autism Spectrum Disorder OR Autism OR Aspergers Disease OR Aspergers Syndrome OR Asperger's Disease OR Asperger's Syndrome OR Kanner's Syndrome OR Kanner Syndrome OR Kanners Syndrome OR Schizophrenia OR Catatonic Schizophrenia OR Disorganized Schizophrenia OR Paranoid Schizophrenia

OR Psychotic Disorder OR Psychotic Disorders OR Psychosis OR Psychoses OR Schizoaffective Disorder OR Schizoaffective Disorders OR Schizophreniform Disorders OR Schizophreniform Disorder OR Psychotic Affective Disorder OR Psychotic Affective Disorders OR Psychotic Mood Disorders OR Psychotic Mood Disorder OR Affective Psychoses OR Post-Traumatic Stress Disorder OR Post Traumatic Stress Disorder OR Post-Traumatic Stress Disorders OR Post Traumatic Stress Disorders OR Traumatic Stress Disorder OR Traumatic Stress Disorders OR Stress Disorder OR Stress Disorders OR Epilepsy OR Epilepsies OR Epileptic
"Mental Health Services"[Mesh] OR "Psychiatry/supply and distribution"[Mesh] OR "Psychiatry/organization and administration"[Mesh] OR "Psychiatry/therapy"[Mesh] OR "Psychiatry/utilization"[Mesh] OR "Emergency Services, Psychiatric"[Mesh] OR "Mental Disorders/prevention and control"[Mesh] OR "Mental Disorders/organization and administration"[Mesh] OR "Mental Disorders/economics"[Mesh] OR "Mental Disorders/legislation and jurisprudence"[Mesh] OR "Mental Disorders/therapy"[Mesh] OR "Mental Disorders/statistics and numerical data"[Mesh] OR "Mental Disorders/trends"[Mesh] OR "Epilepsy/prevention and control"[Mesh] OR "Epilepsy/organization and administration"[Mesh] OR "Epilepsy/economics"[Mesh] OR "Epilepsy/legislation and jurisprudence"[Mesh] OR "Epilepsy/therapy"[Mesh] OR "Epilepsy/statistics and numerical data"[Mesh]

Low and Middle Income Country Terms
Afghanistan OR Albania OR Algeria OR Angola OR Antigua OR Argentina OR Armenia OR Azerbaijan OR Bangladesh OR Barbados OR Belarus OR Belize OR Benin OR Bhutan OR Bolivia OR Bosnia OR Botswana OR Brazil OR Bulgaria OR Burkina Faso OR Burundi OR Cabo Verde OR Cambodia OR Cameroon OR Central African Republic OR Chad OR Chile OR China OR Colombia OR Comoros OR Congo OR Costa Rica OR Cote D'Ivoire OR Côte d'Ivoire OR Cuba OR Cyprus OR Democratic People's Republic of Korea OR Democratic Peoples Republic of Korea OR Deprived Countries OR Deprived Population OR Deprived Populations OR Developing Countries OR Developing Country OR Developing Economies OR Developing Economy OR Developing Nation OR Developing Nations OR Developing Population OR Developing Populations OR Developing World OR Djibouti OR Dominica OR Dominican Republic OR Ecuador OR Egypt OR El Salvador OR Equatorial Guinea OR Eritrea OR Ethiopia OR Fiji OR Gabon OR Gambia OR Gaza OR Georgia OR Ghana OR Gibraltar OR Greece OR Grenada OR Guatemala OR Guinea OR Guinea-Bissau OR Guyana OR Haiti OR Honduras OR Hungary OR India OR Indonesia OR Iran OR Iraq OR Ivory Coast OR Jamaica OR Jordan OR Kazakhstan OR Kenya OR Kiribati OR Kosovo OR Kyrgyz Republic OR LAMI Countries OR LAMI Country OR LAMIC OR Lao OR Lebanon OR Lesotho OR Less Developed Countries OR Less Developed Country OR Less Developed Economies OR Less Developed Nation OR Less Developed Nations OR Less Developed World OR Lesser Developed Countries OR Lesser Developed Nations OR Liberia OR Libya OR LMIC OR LMICS OR Low GDP OR Low GNP OR Low Gross Domestic OR Low Gross National OR Low Income Countries OR Low Income Country OR Low Income Economies OR Low Income Economy OR Low Income Nations OR Low Income Population OR Low Income Populations OR Lower GDP OR lower gross domestic OR Lower Income Countries OR Lower Income Country OR Lower Income Nations OR Lower Income Population OR Lower Income Populations OR Macao OR Macedonia OR Madagascar OR Malawi OR Malaysia OR Maldives OR Mali OR Malta OR Marshall Islands OR Mauritania OR Mauritius OR Mexico OR Micronesia OR Middle Income Countries OR Middle Income Country OR Middle Income Economies OR Middle Income Nation OR Middle Income Nations OR Middle Income Population OR Middle Income Populations OR Moldova OR Mongolia OR Montenegro OR Morocco OR Mozambique OR Myanmar OR Namibia OR Nauru OR Nepal OR New Caledonia OR Nicaragua OR Niger OR Nigeria OR North Korea OR Oman OR Pakistan OR Panama OR Papua New Guinea OR Paraguay OR Peru OR Philippines OR Poland OR Poor Countries OR Poor Country OR Poor Economies OR Poor Economy OR Poor Nation OR Poor Nations OR Poor Population OR Poor Populations OR poor world OR Poorer Countries OR Poorer Economies OR Poorer Economy OR Poorer Nations OR Poorer Population OR Poorer Populations OR Portugal OR Puerto Rico OR Republic of Congo OR Republic of Korea OR Romania OR Russian Federation OR Rwanda OR Samoa OR Sao Tome OR São Tomé OR Senegal OR Serbia OR Seychelles OR Sierra Leone OR Solomon Islands OR Somalia OR South Africa OR South Korea OR South Sudan OR Sri Lanka OR St. Kitts OR St. Lucia OR St. Vincent OR Sudan OR Suriname OR Swaziland OR Syria OR Tajikistan OR Tanzania OR Thailand OR

the Grenadines OR Third World OR Timor-Leste OR Tobago OR Togo OR Tonga OR Transitional Countries OR Transitional Country OR Transitional Economies OR Transitional Economy OR Trinidad OR Tunisia OR Turkey OR Turkmenistan OR Tuvalu OR Uganda OR Ukraine OR Under Developed Countries OR Under Developed Country OR under developed nations OR Under Developed World OR Under Served Population OR Under Served Populations OR Underdeveloped Countries OR Underdeveloped Country OR underdeveloped economies OR underdeveloped nations OR underdeveloped population OR Underdeveloped World OR Underserved Countries OR Underserved Nations OR Underserved Population OR Underserved Populations OR Uruguay OR Uzbekistan OR Vanuatu OR Venezuela OR Vietnam OR West Bank OR Yemen OR Zambia OR Zimbabwe

Health Insurance Search String	
(Title/Abstract) OR (Mesh)	National Health Insurance[Title/Abstract] OR Social Health Insurance[Title/Abstract] OR Community-based Health Insurance[Title/Abstract] OR Community based Health Insurance[Title/Abstract] OR Community Health Insurance[Title/Abstract] OR Mandatory Health Insurance[Title/Abstract] OR Informal Health Insurance[Title/Abstract] OR Single-payer system[Title/Abstract] OR Single Payer System[Title/Abstract] OR
	“National Health Programs/economics"[Mesh] OR "Insurance"[Mesh] OR "Health Care Sector/economics"[Mesh] OR "Health Care Sector/organization and administration"[Mesh] OR "Financing, Organized"[Mesh] OR "Healthcare Financing"[Mesh] OR "Health Equity/economics"[Mesh] OR "Universal Coverage/economics"[Mesh] OR "Health Care Reform/economics"[Mesh] OR “National Health Programs/legislation and jurisprudence”[Mesh] OR “Nsational Health Programs/organization and administration”[Mesh] OR “Universal Coverage/legislation and jurisprudence”[Mesh]

Mental Healthcare Utilization Search String	
(All Fields) OR (Mesh)	(Mental Health Services OR Mental Health Service OR Mental Healthcare OR Mental Health Care OR Mental Health System OR Psychiatric Services OR Psychiatric Care OR Psychiatry Services OR Psychiatric Health Care OR Psychiatric Healthcare OR Psychiatry OR Mental Illness OR Mental Health OR Severe Mental Disorder OR Severe Mental Disorders OR Common Mental Disorder OR Common Mental Disorders OR Mental Illness OR Mentally Ill Persons OR Substance Disorder OR Substance Disorders OR Substance Abuse OR Substance-Use Disorder OR Substance Use Disorder OR Substance-Use Disorders OR Substance Use Disorders OR Alcohol Use Disorder OR Alcohol Use Disorders OR Alcohol Abuse OR Alcohol-Related Disorder OR Alcohol Related Disorder OR Alcohol Addiction OR Opioid Abuse OR Opiate Addiction OR Opioid-Related Disorder OR Opioid Related Disorder OR Cannabis-Related Disorder OR Cannabis Related Disorder OR Cocaine Related Disorder OR Cocaine-Related Disorder OR Cocaine Addiction OR Amphetamine-Related Disorder OR Amphetamine Related Disorder OR Amphetamine Addiction OR Heroin Dependence OR Heroin Abuse OR Heroin Addiction OR Substance Induced Psychoses OR Substance-Induced Psychoses OR Anxiety Disorders OR Anxiety Disorder OR Bipolar Disorder OR Bipolar Disorders OR Manic-Depressive Psychosis OR Manic Depressive Psychosis OR Bipolar Affective Psychosis OR Bipolar Affective Psychosis OR Manic-Depressive Psychoses OR Manic Depressive Psychoses OR Bipolar Affective Psychoses OR Bipolar Affective Psychoses OR Bipolar Depression OR Anorexia Nervosa OR Binge-Eating Disorder OR Bulimia Nervosa OR Anorexia OR Binge Eating OR Binge-Eating OR Bulimia OR Depressive Disorders OR Depressive Disorder OR Depression OR Unipolar Depression OR Unipolar Depressions OR Mood Disorder OR Mood Disorders OR Dementia OR Dementias OR Attention Deficit Disorders OR Attention Deficit Disorders OR Conduct Disorder OR Conduct Disorders OR Neurocognitive Disorder OR Neurotic Disorder OR Neurodevelopmental Disorder OR Developmental Disability OR Development Disability OR Developmental Disorder OR Development Disorder OR Autism Spectrum Disorder OR Asperger Syndrome OR Autistic Disorder OR Autistic Disorder OR Autism Spectrum Disorders OR Autism Spectrum Disorder OR Autism OR Aspergers Disease OR Aspergers Syndrome OR Asperger's Disease OR Asperger's Syndrome OR Kanner's Syndrome OR Kanner Syndrome OR Kanners Syndrome OR Schizophrenia OR Catatonic Schizophrenia OR Disorganized Schizophrenia OR Paranoid Schizophrenia OR Psychotic Disorder OR Psychotic Disorders OR Psychosis OR Psychoses OR Schizoaffective Disorder OR Schizoaffective Disorders OR Schizophreniform Disorders OR Schizophreniform Disorder OR Psychotic Affective Disorder OR Psychotic Affective Disorders OR Psychotic Mood Disorders OR Psychotic Mood Disorder OR Affective Psychoses OR Post-Traumatic Stress Disorder OR Post Traumatic Stress Disorder OR Post-Traumatic Stress Disorders OR Post Traumatic Stress Disorders OR Traumatic Stress Disorder OR Traumatic Stress Disorders OR Stress Disorder OR Stress Disorders OR Epilepsy OR Epilepsies OR Epileptic)
	("Mental Health Services"[Mesh] OR "Psychiatry/supply and distribution"[Mesh] OR "Psychiatry/organization and administration"[Mesh] OR "Psychiatry/therapy"[Mesh] OR "Psychiatry/utilization"[Mesh] OR "Emergency Services, Psychiatric"[Mesh] OR "Mental

	Disorders/prevention and control"[Mesh] OR "Mental Disorders/organization and administration"[Mesh] OR "Mental Disorders/economics"[Mesh] OR "Mental Disorders/legislation and jurisprudence"[Mesh] OR "Mental Disorders/therapy"[Mesh] OR "Mental Disorders/statistics and numerical data"[Mesh] OR "Mental Disorders/trends"[Mesh] OR "Epilepsy/prevention and control"[Mesh] OR "Epilepsy/organization and administration"[Mesh] OR "Epilepsy/economics"[Mesh] OR "Epilepsy/legislation and jurisprudence"[Mesh] OR "Epilepsy/therapy"[Mesh] OR "Epilepsy/statistics and numerical data"[Mesh])

Low and Middle Income Country Search String	
(Title/Abstract)	(Afghanistan[Title/Abstract] OR Albania[Title/Abstract] OR Algeria[Title/Abstract] OR Angola[Title/Abstract] OR Antigua[Title/Abstract] OR Argentina[Title/Abstract] OR Armenia[Title/Abstract] OR Azerbaijan[Title/Abstract] OR Bangladesh[Title/Abstract] OR Barbados[Title/Abstract] OR Belarus[Title/Abstract] OR Belize[Title/Abstract] OR Benin[Title/Abstract] OR Bhutan[Title/Abstract] OR Bolivia[Title/Abstract] OR Bosnia[Title/Abstract] OR Botswana[Title/Abstract] OR Brazil[Title/Abstract] OR Bulgaria[Title/Abstract] OR Burkina Faso[Title/Abstract] OR Burundi[Title/Abstract] OR Cabo Verde[Title/Abstract] OR Cambodia[Title/Abstract] OR Cameroon[Title/Abstract] OR Central African Republic[Title/Abstract] OR Chad[Title/Abstract] OR Chile[Title/Abstract] OR China[Title/Abstract] OR Colombia[Title/Abstract] OR Comoros[Title/Abstract] OR Congo[Title/Abstract] OR Costa Rica[Title/Abstract] OR Cote D'Ivoire[Title/Abstract] OR Côte d'Ivoire[Title/Abstract] OR Cuba[Title/Abstract] OR Cyprus[Title/Abstract] OR Democratic People's Republic of Korea[Title/Abstract] OR Democratic Peoples Republic of Korea[Title/Abstract] OR Deprived Countries[Title/Abstract] OR Deprived Population[Title/Abstract] OR Deprived Populations[Title/Abstract] OR Developing Countries[Title/Abstract] OR Developing Country[Title/Abstract] OR Developing Economies[Title/Abstract] OR Developing Economy[Title/Abstract] OR Developing Nation[Title/Abstract] OR Developing Nations[Title/Abstract] OR Developing Population[Title/Abstract] OR Developing Populations[Title/Abstract] OR Developing World[Title/Abstract] OR Djibouti[Title/Abstract] OR Dominica[Title/Abstract] OR Dominican Republic[Title/Abstract] OR Ecuador[Title/Abstract] OR Egypt[Title/Abstract] OR El Salvador[Title/Abstract] OR Equatorial Guinea[Title/Abstract] OR Eritrea[Title/Abstract] OR Ethiopia[Title/Abstract] OR Fiji[Title/Abstract] OR Gabon[Title/Abstract] OR Gambia[Title/Abstract] OR Gaza[Title/Abstract] OR Georgia[Title/Abstract] OR Ghana[Title/Abstract] OR Gibraltar[Title/Abstract] OR Greece[Title/Abstract] OR Grenada[Title/Abstract] OR Guatemala[Title/Abstract] OR Guinea[Title/Abstract] OR Guinea-Bissau[Title/Abstract] OR Guyana[Title/Abstract] OR Haiti[Title/Abstract] OR Honduras[Title/Abstract] OR Hungary[Title/Abstract] OR India[Title/Abstract] OR Indonesia[Title/Abstract] OR Iran[Title/Abstract] OR Iraq[Title/Abstract] OR Ivory Coast[Title/Abstract] OR Jamaica[Title/Abstract] OR Jordan[Title/Abstract] OR Kazakhstan[Title/Abstract] OR Kenya[Title/Abstract] OR Kiribati[Title/Abstract] OR Kosovo[Title/Abstract] OR Kyrgyz Republic[Title/Abstract] OR LAMI Countries[Title/Abstract] OR LAMI Country[Title/Abstract] OR LAMIC[Title/Abstract] OR Lao[Title/Abstract] OR Lebanon[Title/Abstract] OR Lesotho[Title/Abstract] OR Less Developed Countries[Title/Abstract] OR Less Developed Country[Title/Abstract] OR Less Developed Economies [Title/Abstract] OR Less Developed Nation[Title/Abstract] OR Less Developed Nations[Title/Abstract] OR Less Developed World[Title/Abstract] OR Lesser Developed Countries[Title/Abstract] OR Lesser Developed Nations[Title/Abstract] OR Liberia[Title/Abstract] OR Libya[Title/Abstract] OR LMIC[Title/Abstract] OR LMICS[Title/Abstract] OR Low GDP[Title/Abstract] OR Low GNP[Title/Abstract] OR Low Gross Domestic[Title/Abstract] OR Low Gross National[Title/Abstract] OR Low Income Countries[Title/Abstract] OR Low Income Country[Title/Abstract] OR Low Income Economies [Title/Abstract] OR Low Income Economy[Title/Abstract] OR Low Income Nations[Title/Abstract] OR Low Income Population[Title/Abstract] OR Low Income Populations[Title/Abstract] OR Lower

	GDP[Title/Abstract] OR lower gross domestic[Title/Abstract] OR Lower Income Countries[Title/Abstract] OR Lower Income Country[Title/Abstract] OR Lower Income Nations[Title/Abstract] OR Lower Income Population[Title/Abstract] OR Lower Income Populations[Title/Abstract] OR Macao[Title/Abstract] OR Macedonia[Title/Abstract] OR Madagascar[Title/Abstract] OR Malawi[Title/Abstract] OR Malaysia[Title/Abstract] OR Maldives[Title/Abstract] OR Mali[Title/Abstract] OR Malta[Title/Abstract] OR Marshall Islands[Title/Abstract] OR Mauritania[Title/Abstract] OR Mauritius[Title/Abstract] OR Mexico[Title/Abstract] OR Micronesia[Title/Abstract] OR Middle Income Countries[Title/Abstract] OR Middle Income Country[Title/Abstract] OR Middle Income Economies [Title/Abstract] OR Middle Income Nation[Title/Abstract] OR Middle Income Nations[Title/Abstract] OR Middle Income Population[Title/Abstract] OR Middle Income Populations[Title/Abstract] OR Moldova[Title/Abstract] OR Mongolia[Title/Abstract] OR Montenegro[Title/Abstract] OR Morocco[Title/Abstract] OR Mozambique[Title/Abstract] OR Myanmar[Title/Abstract] OR Namibia[Title/Abstract] OR Nauru[Title/Abstract] OR Nepal[Title/Abstract] OR New Caledonia[Title/Abstract] OR Nicaragua[Title/Abstract] OR Niger[Title/Abstract] OR Nigeria[Title/Abstract] OR North Korea[Title/Abstract] OR Oman[Title/Abstract] OR Pakistan[Title/Abstract] OR Panama[Title/Abstract] OR Papua New Guinea[Title/Abstract] OR Paraguay[Title/Abstract] OR Peru[Title/Abstract] OR Philippines[Title/Abstract] OR Poland[Title/Abstract] OR Poor Countries[Title/Abstract] OR Poor Country[Title/Abstract] OR Poor Economies [Title/Abstract] OR Poor Economy[Title/Abstract] OR Poor Nation[Title/Abstract] OR Poor Nations[Title/Abstract] OR Poor Population[Title/Abstract] OR Poor Populations[Title/Abstract] OR poor world[Title/Abstract] OR Poorer Countries[Title/Abstract] OR Poorer Economies [Title/Abstract] OR Poorer Economy[Title/Abstract] OR Poorer Nations[Title/Abstract] OR Poorer Population[Title/Abstract] OR Poorer Populations[Title/Abstract] OR Portugal[Title/Abstract] OR Puerto Rico[Title/Abstract] OR Republic of Congo[Title/Abstract] OR Republic of Korea[Title/Abstract] OR Romania[Title/Abstract] OR Russian Federation[Title/Abstract] OR Rwanda[Title/Abstract] OR Samoa[Title/Abstract] OR Sao Tome[Title/Abstract] OR São Tomé[Title/Abstract] OR Senegal[Title/Abstract] OR Serbia[Title/Abstract] OR Seychelles[Title/Abstract] OR Sierra Leone[Title/Abstract] OR Solomon Islands[Title/Abstract] OR Somalia[Title/Abstract] OR South Africa[Title/Abstract] OR South Korea[Title/Abstract] OR South Sudan[Title/Abstract] OR Sri Lanka[Title/Abstract] OR St. Kitts[Title/Abstract] OR St. Lucia[Title/Abstract] OR St. Vincent [Title/Abstract] OR Sudan[Title/Abstract] OR Suriname[Title/Abstract] OR Swaziland[Title/Abstract] OR Syria[Title/Abstract] OR Tajikistan[Title/Abstract] OR Tanzania[Title/Abstract] OR Thailand[Title/Abstract] OR the Grenadines[Title/Abstract] OR Third World[Title/Abstract] OR Timor-Leste[Title/Abstract] OR Tobago[Title/Abstract] OR Togo[Title/Abstract] OR Tonga[Title/Abstract] OR Transitional Countries[Title/Abstract] OR Transitional Country[Title/Abstract] OR Transitional Economies[Title/Abstract] OR Transitional Economy[Title/Abstract] OR Trinidad[Title/Abstract] OR Tunisia[Title/Abstract] OR Turkey[Title/Abstract] OR Turkmenistan[Title/Abstract] OR Tuvalu[Title/Abstract] OR Uganda[Title/Abstract] OR Ukraine[Title/Abstract] OR Under Developed Countries[Title/Abstract] OR Under Developed Country[Title/Abstract] OR under developed nations[Title/Abstract] OR Under Developed World[Title/Abstract] OR Under Served Population[Title/Abstract] OR Under Served Populations[Title/Abstract] OR Underdeveloped Countries[Title/Abstract] OR Underdeveloped Country[Title/Abstract] OR underdeveloped economies[Title/Abstract] OR underdeveloped nations[Title/Abstract] OR underdeveloped population[Title/Abstract] OR Underdeveloped World[Title/Abstract] OR Underserved Countries[Title/Abstract] OR Underserved Nations[Title/Abstract] OR Underserved Population[Title/Abstract] OR Underserved Populations[Title/Abstract] OR Uruguay[Title/Abstract] OR Uzbekistan[Title/Abstract] OR Vanuatu[Title/Abstract] OR Venezuela[Title/Abstract] OR Vietnam[Title/Abstract] OR West Bank[Title/Abstract] OR Yemen[Title/Abstract] OR Zambia[Title/Abstract] OR Zimbabwe[Title/Abstract])

Limits (Not High Income Countries) Search String

NOT (Mesh)	“Aruba"[Mesh] OR "Australia"[Mesh] OR "South Australia"[Mesh] OR "Western Australia"[Mesh] OR "Austria"[Mesh] OR "Bahamas"[Mesh] OR "Bahrain"[Mesh] OR "Belgium"[Mesh] OR "Bermuda"[Mesh] OR "Brunei"[Mesh] OR "Canada"[Mesh] OR "Channel Islands"[Mesh] OR "Denmark"[Mesh] OR "Finland"[Mesh] OR "France"[Mesh] OR "Germany"[Mesh] OR "Germany, West"[Mesh] OR "Germany, East"[Mesh] OR "Greenland"[Mesh] OR "Guam"[Mesh] OR "Hong Kong"[Mesh] OR "Iceland"[Mesh] OR "Ireland"[Mesh] OR "United Kingdom"[Mesh] OR "Israel"[Mesh] OR "Italy"[Mesh] OR "Japan"[Mesh] OR "Kuwait"[Mesh] OR "Luxembourg"[Mesh] OR "Netherlands"[Mesh] OR "New Zealand"[Mesh] OR "Norway"[Mesh] OR "Qatar"[Mesh] OR "Saudi Arabia"[Mesh] OR "Singapore"[Mesh] OR "Spain"[Mesh] OR "Sweden"[Mesh] OR "Switzerland"[Mesh] OR "Taiwan"[Mesh] OR "United Arab Emirates"[Mesh] OR "United States"[Mesh] OR "United States Virgin Islands"[Mesh]

**FULL SEARCH STRING: PubMed (October 03, 2018) Yield: 796**
(((((((National Health Insurance[Title/Abstract] OR Social Health Insurance[Title/Abstract] OR Community-based Health Insurance[Title/Abstract] OR Community based Health Insurance[Title/Abstract] OR Community Health Insurance[Title/Abstract] OR Mandatory Health Insurance[Title/Abstract] OR Informal Health Insurance[Title/Abstract] OR Single-payer system[Title/Abstract] OR Single Payer System[Title/Abstract]))) OR (("National Health Programs/economics"[Mesh] OR "Insurance"[Mesh] OR "Health Care Sector/economics"[Mesh] OR "Health Care Sector/organization and administration"[Mesh] OR "Financing, Organized"[Mesh] OR "Healthcare Financing"[Mesh] OR "Health Equity/economics"[Mesh] OR "Universal Coverage/economics"[Mesh] OR "Health Care Reform/economics"[Mesh] OR “National Health Programs/legislation and jurisprudence”[Mesh] OR “National Health Programs/organization and administration”[Mesh] OR “Universal Coverage/legislation and jurisprudence”[Mesh])))) AND ((((Mental Health Services OR Mental Health Service OR Mental Healthcare OR Mental Health Care OR Mental Health System OR Psychiatric Services OR Psychiatric Care OR Psychiatry Services OR Psychiatric Health Care OR Psychiatric Healthcare OR Psychiatry OR Mental Illness OR Mental Health OR Severe Mental Disorder OR Severe Mental Disorders OR Common Mental Disorder OR Common Mental Disorders OR Mental Illness OR Mentally Ill Persons OR Substance Disorder OR Substance Disorders OR Substance Abuse OR Substance-Use Disorder OR Substance Use Disorder OR Substance-Use Disorders OR Substance Use Disorders OR Alcohol Use Disorder OR Alcohol Use Disorders OR Alcohol Abuse OR Alcohol-Related Disorder OR Alcohol Related Disorder OR Alcohol Addiction OR Opioid Abuse OR Opiate Addiction OR Opioid-Related Disorder OR Opioid Related Disorder OR Cannabis-Related Disorder OR Cannabis Related Disorder OR Cocaine Related Disorder OR Cocaine-Related Disorder OR Cocaine Addiction OR Amphetamine-Related Disorder OR Amphetamine Related Disorder OR Amphetamine Addiction OR Heroin Dependence OR Heroin Abuse OR Heroin Addiction OR Substance Induced Psychoses OR Substance-Induced Psychoses OR Anxiety Disorders OR Anxiety Disorder OR Bipolar Disorder OR Bipolar Disorders OR Manic-Depressive Psychosis OR Manic Depressive Psychosis OR Bipolar Affective Psychosis OR Bipolar Affective Psychosis OR Manic-Depressive Psychoses OR Manic Depressive Psychoses OR Bipolar Affective Psychoses OR Bipolar Affective Psychoses OR Bipolar Depression OR Anorexia Nervosa OR Binge-Eating Disorder OR Bulimia Nervosa OR Anorexia OR Binge Eating OR Binge-Eating OR Bulimia OR Depressive Disorders OR Depressive Disorder OR Depression OR Unipolar Depression OR Unipolar Depressions OR Mood Disorder OR Mood Disorders OR Dementia OR Dementias OR Attention Deficit Disorders OR Attention Deficit Disorders OR Conduct Disorder OR Conduct Disorders OR Neurocognitive Disorder OR Neurotic Disorder OR Neurodevelopmental Disorder OR Developmental Disability OR Development Disability OR Developmental Disorder OR Development Disorder OR Autism Spectrum Disorder OR Asperger Syndrome OR Autistic Disorder OR Autistic Disorder OR Autism Spectrum Disorders OR Autism Spectrum Disorder OR Autism OR Aspergers Disease OR Aspergers Syndrome OR Asperger's Disease OR Asperger's Syndrome OR Kanner's Syndrome OR Kanner Syndrome OR Kanners Syndrome OR Schizophrenia OR Catatonic Schizophrenia OR Disorganized Schizophrenia OR Paranoid Schizophrenia OR Psychotic Disorder OR Psychotic Disorders OR Psychosis OR Psychoses OR Schizoaffective Disorder OR Schizoaffective Disorders OR Schizophreniform Disorders OR Schizophreniform Disorder OR

Psychotic Affective Disorder OR Psychotic Affective Disorders OR Psychotic Mood Disorders OR Psychotic Mood Disorder OR Affective Psychoses OR Post-Traumatic Stress Disorder OR Post Traumatic Stress Disorder OR Post-Traumatic Stress Disorders OR Post Traumatic Stress Disorders OR Traumatic Stress Disorder OR Traumatic Stress Disorders OR Stress Disorder OR Stress Disorders OR Epilepsy OR Epilepsies OR Epileptic))) OR (("Mental Health Services"[Mesh] OR "Psychiatry/supply and distribution"[Mesh] OR "Psychiatry/organization and administration"[Mesh] OR "Psychiatry/therapy"[Mesh] OR "Psychiatry/utilization"[Mesh] OR "Emergency Services, Psychiatric"[Mesh] OR "Mental Disorders/prevention and control"[Mesh] OR "Mental Disorders/organization and administration"[Mesh] OR "Mental Disorders/economics"[Mesh] OR "Mental Disorders/legislation and jurisprudence"[Mesh] OR "Mental Disorders/therapy"[Mesh] OR "Mental Disorders/statistics and numerical data"[Mesh] OR "Mental Disorders/trends"[Mesh] OR "Epilepsy/prevention and control"[Mesh] OR "Epilepsy/organization and administration"[Mesh] OR "Epilepsy/economics"[Mesh] OR "Epilepsy/legislation and jurisprudence"[Mesh] OR "Epilepsy/therapy"[Mesh] OR "Epilepsy/statistics and numerical data"[Mesh])))) AND ((Afghanistan[Title/Abstract] OR Albania[Title/Abstract] OR Algeria[Title/Abstract] OR Angola[Title/Abstract] OR Antigua[Title/Abstract] OR Argentina[Title/Abstract] OR Armenia[Title/Abstract] OR Azerbaijan[Title/Abstract] OR Bangladesh[Title/Abstract] OR Barbados[Title/Abstract] OR Belarus[Title/Abstract] OR Belize[Title/Abstract] OR Benin[Title/Abstract] OR Bhutan[Title/Abstract] OR Bolivia[Title/Abstract] OR Bosnia[Title/Abstract] OR Botswana[Title/Abstract] OR Brazil[Title/Abstract] OR Bulgaria[Title/Abstract] OR Burkina Faso[Title/Abstract] OR Burundi[Title/Abstract] OR Cabo Verde[Title/Abstract] OR Cambodia[Title/Abstract] OR Cameroon[Title/Abstract] OR Central African Republic[Title/Abstract] OR Chad[Title/Abstract] OR Chile[Title/Abstract] OR China[Title/Abstract] OR Colombia[Title/Abstract] OR Comoros[Title/Abstract] OR Congo[Title/Abstract] OR Costa Rica[Title/Abstract] OR Cote D'Ivoire[Title/Abstract] OR Côte d'Ivoire[Title/Abstract] OR Cuba[Title/Abstract] OR Cyprus[Title/Abstract] OR Democratic People's Republic of Korea[Title/Abstract] OR Democratic Peoples Republic of Korea[Title/Abstract] OR Deprived Countries[Title/Abstract] OR Deprived Population[Title/Abstract] OR Deprived Populations[Title/Abstract] OR Developing Countries[Title/Abstract] OR Developing Country[Title/Abstract] OR Developing Economies[Title/Abstract] OR Developing Economy[Title/Abstract] OR Developing Nation[Title/Abstract] OR Developing Nations[Title/Abstract] OR Developing Population[Title/Abstract] OR Developing Populations[Title/Abstract] OR Developing World[Title/Abstract] OR Djibouti[Title/Abstract] OR Dominica[Title/Abstract] OR Dominican Republic[Title/Abstract] OR Ecuador[Title/Abstract] OR Egypt[Title/Abstract] OR El Salvador[Title/Abstract] OR Equatorial Guinea[Title/Abstract] OR Eritrea[Title/Abstract] OR Ethiopia[Title/Abstract] OR Fiji[Title/Abstract] OR Gabon[Title/Abstract] OR Gambia[Title/Abstract] OR Gaza[Title/Abstract] OR Georgia[Title/Abstract] OR Ghana[Title/Abstract] OR Gibraltar[Title/Abstract] OR Greece[Title/Abstract] OR Grenada[Title/Abstract] OR Guatemala[Title/Abstract] OR Guinea[Title/Abstract] OR Guinea-Bissau[Title/Abstract] OR Guyana[Title/Abstract] OR Haiti[Title/Abstract] OR Honduras[Title/Abstract] OR Hungary[Title/Abstract] OR India[Title/Abstract] OR Indonesia[Title/Abstract] OR Iran[Title/Abstract] OR Iraq[Title/Abstract] OR Ivory Coast[Title/Abstract] OR Jamaica[Title/Abstract] OR Jordan[Title/Abstract] OR Kazakhstan[Title/Abstract] OR Kenya[Title/Abstract] OR Kiribati[Title/Abstract] OR Kosovo[Title/Abstract] OR Kyrgyz Republic[Title/Abstract] OR LAMI Countries[Title/Abstract] OR LAMI Country[Title/Abstract] OR LAMIC[Title/Abstract] OR Lao[Title/Abstract] OR Lebanon[Title/Abstract] OR Lesotho[Title/Abstract] OR Less Developed Countries[Title/Abstract] OR Less Developed Country[Title/Abstract] OR Less Developed Economies [Title/Abstract] OR Less Developed Nation[Title/Abstract] OR Less Developed Nations[Title/Abstract] OR Less Developed World[Title/Abstract] OR Lesser Developed Countries[Title/Abstract] OR Lesser Developed Nations[Title/Abstract] OR Liberia[Title/Abstract] OR Libya[Title/Abstract] OR LMIC[Title/Abstract] OR LMICS[Title/Abstract] OR Low GDP[Title/Abstract] OR Low GNP[Title/Abstract] OR Low Gross Domestic[Title/Abstract] OR Low Gross National[Title/Abstract] OR Low Income Countries[Title/Abstract] OR Low Income Country[Title/Abstract] OR Low Income Economies [Title/Abstract] OR Low Income Economy[Title/Abstract] OR Low Income Nations[Title/Abstract] OR Low Income Population[Title/Abstract] OR Low Income Populations[Title/Abstract] OR Lower

GDP[Title/Abstract] OR lower gross domestic[Title/Abstract] OR Lower Income Countries[Title/Abstract] OR Lower Income Country[Title/Abstract] OR Lower Income Nations[Title/Abstract] OR Lower Income Population[Title/Abstract] OR Lower Income Populations[Title/Abstract] OR Macao[Title/Abstract] OR Macedonia[Title/Abstract] OR Madagascar[Title/Abstract] OR Malawi[Title/Abstract] OR Malaysia[Title/Abstract] OR Maldives[Title/Abstract] OR Mali[Title/Abstract] OR Malta[Title/Abstract] OR Marshall Islands[Title/Abstract] OR Mauritania[Title/Abstract] OR Mauritius[Title/Abstract] OR Mexico[Title/Abstract] OR Micronesia[Title/Abstract] OR Middle Income Countries[Title/Abstract] OR Middle Income Country[Title/Abstract] OR Middle Income Economies [Title/Abstract] OR Middle Income Nation[Title/Abstract] OR Middle Income Nations[Title/Abstract] OR Middle Income Population[Title/Abstract] OR Middle Income Populations[Title/Abstract] OR Moldova[Title/Abstract] OR Mongolia[Title/Abstract] OR Montenegro[Title/Abstract] OR Morocco[Title/Abstract] OR Mozambique[Title/Abstract] OR Myanmar[Title/Abstract] OR Namibia[Title/Abstract] OR Nauru[Title/Abstract] OR Nepal[Title/Abstract] OR New Caledonia[Title/Abstract] OR Nicaragua[Title/Abstract] OR Niger[Title/Abstract] OR Nigeria[Title/Abstract] OR North Korea[Title/Abstract] OR Oman[Title/Abstract] OR Pakistan[Title/Abstract] OR Panama[Title/Abstract] OR Papua New Guinea[Title/Abstract] OR Paraguay[Title/Abstract] OR Peru[Title/Abstract] OR Philippines[Title/Abstract] OR Poland[Title/Abstract] OR Poor Countries[Title/Abstract] OR Poor Country[Title/Abstract] OR Poor Economies [Title/Abstract] OR Poor Economy[Title/Abstract] OR Poor Nation[Title/Abstract] OR Poor Nations[Title/Abstract] OR Poor Population[Title/Abstract] OR Poor Populations[Title/Abstract] OR poor world[Title/Abstract] OR Poorer Countries[Title/Abstract] OR Poorer Economies [Title/Abstract] OR Poorer Economy[Title/Abstract] OR Poorer Nations[Title/Abstract] OR Poorer Population[Title/Abstract] OR Poorer Populations[Title/Abstract] OR Portugal[Title/Abstract] OR Puerto Rico[Title/Abstract] OR Republic of Congo[Title/Abstract] OR Republic of Korea[Title/Abstract] OR Romania[Title/Abstract] OR Russian Federation[Title/Abstract] OR Rwanda[Title/Abstract] OR Samoa[Title/Abstract] OR Sao Tome[Title/Abstract] OR São Tomé[Title/Abstract] OR Senegal[Title/Abstract] OR Serbia[Title/Abstract] OR Seychelles[Title/Abstract] OR Sierra Leone[Title/Abstract] OR Solomon Islands[Title/Abstract] OR Somalia[Title/Abstract] OR South Africa[Title/Abstract] OR South Korea[Title/Abstract] OR South Sudan[Title/Abstract] OR Sri Lanka[Title/Abstract] OR St. Kitts[Title/Abstract] OR St. Lucia[Title/Abstract] OR St. Vincent [Title/Abstract] OR Sudan[Title/Abstract] OR Suriname[Title/Abstract] OR Swaziland[Title/Abstract] OR Syria[Title/Abstract] OR Tajikistan[Title/Abstract] OR Tanzania[Title/Abstract] OR Thailand[Title/Abstract] OR the Grenadines[Title/Abstract] OR Third World[Title/Abstract] OR Timor-Leste[Title/Abstract] OR Tobago[Title/Abstract] OR Togo[Title/Abstract] OR Tonga[Title/Abstract] OR Transitional Countries[Title/Abstract] OR Transitional Country[Title/Abstract] OR Transitional Economies[Title/Abstract] OR Transitional Economy[Title/Abstract] OR Trinidad[Title/Abstract] OR Tunisia[Title/Abstract] OR Turkey[Title/Abstract] OR Turkmenistan[Title/Abstract] OR Tuvalu[Title/Abstract] OR Uganda[Title/Abstract] OR Ukraine[Title/Abstract] OR Under Developed Countries[Title/Abstract] OR Under Developed Country[Title/Abstract] OR under developed nations[Title/Abstract] OR Under Developed World[Title/Abstract] OR Under Served Population[Title/Abstract] OR Under Served Populations[Title/Abstract] OR Underdeveloped Countries[Title/Abstract] OR Underdeveloped Country[Title/Abstract] OR underdeveloped economies[Title/Abstract] OR underdeveloped nations[Title/Abstract] OR underdeveloped population[Title/Abstract] OR Underdeveloped World[Title/Abstract] OR Underserved Countries[Title/Abstract] OR Underserved Nations[Title/Abstract] OR Underserved Population[Title/Abstract] OR Underserved Populations[Title/Abstract] OR Uruguay[Title/Abstract] OR Uzbekistan[Title/Abstract] OR Vanuatu[Title/Abstract] OR Venezuela[Title/Abstract] OR Vietnam[Title/Abstract] OR West Bank[Title/Abstract] OR Yemen[Title/Abstract] OR Zambia[Title/Abstract] OR Zimbabwe[Title/Abstract]))) NOT ((“Aruba"[Mesh] OR "Australia"[Mesh] OR "South Australia"[Mesh] OR "Western Australia"[Mesh] OR "Austria"[Mesh] OR "Bahamas"[Mesh] OR "Bahrain"[Mesh] OR "Belgium"[Mesh] OR "Bermuda"[Mesh] OR "Brunei"[Mesh] OR "Canada"[Mesh] OR "Channel Islands"[Mesh] OR "Denmark"[Mesh] OR "Finland"[Mesh] OR "France"[Mesh] OR "Germany"[Mesh] OR "Germany, West"[Mesh] OR "Germany, East"[Mesh] OR "Greenland"[Mesh] OR

"Guam"[Mesh] OR "Hong Kong"[Mesh] OR "Iceland"[Mesh] OR "Ireland"[Mesh] OR "United Kingdom"[Mesh] OR "Israel"[Mesh] OR "Italy"[Mesh] OR "Japan"[Mesh] OR "Kuwait"[Mesh] OR "Luxembourg"[Mesh] OR "Netherlands"[Mesh] OR "New Zealand"[Mesh] OR "Norway"[Mesh] OR "Qatar"[Mesh] OR "Saudi Arabia"[Mesh] OR "Singapore"[Mesh] OR "Spain"[Mesh] OR "Sweden"[Mesh] OR "Switzerland"[Mesh] OR "Taiwan"[Mesh] OR "United Arab Emirates"[Mesh] OR "United States"[Mesh] OR "United States Virgin Islands"[Mesh]))
