# Supplementary material for: The impact of social, national and community-based health insurance on health care utilization for mental, neurological and substance-use disorders in low- and middle-income countries: a systematic review
Source: Health Econ Rev. 2020 Apr 24;10:11. doi: 10.1186/s13561-020-00268-x (PMC7181535; doi:10.1186/s13561-020-00268-x)
Supplement: Supplementary file 2 — Additional file 2. [file 13561_2020_268_MOESM2_ESM.docx]

| **Additional File 2: Characteristics of the Health Insurance Mechanisms examined by the Included Studies** | | | | | | | | |
| --- | --- | --- | --- | --- | --- | --- | --- | --- |
| **Location** | **Health Insurance Mechanism type** | **Health Insurance Mechanism Name** | **Year of scheme establishment** | **Target Beneficiaries and Coverage** | **Revenue Collection and Pooling** | **Benefits Packages Covered** | **Provider Payment Mechanism** | **Co-payments** |
| Chile | SHI | National Health Fund (FONASA) | 1979 | Category A: the indigent, unemployed or inactive; Category B: very low income; Category C: lower-middle income and D: higher-middle earning group. Category C and D also have the option to use private health care; in 2005, coverage for FONASA was 70% | - Uniform compulsory health insurance contribution of 7% (ceiling of $2000) of salary comprising one third of public funding with the remainder coming from the state. - Between 32-40% of contributions of higher-income public beneficiaries cross-subsidize funding for poorer beneficiaries (progressive) [1]. - Regime of Explicit Health Guarantees (AUGE) reforms implemented in 2002-03 to achieve universal health coverage. | - Users of categories A and B entitled only to services provided by public hospitals and clinics. - Lowest income groups entitled to free care directly from FONASA but only eligible for certain services and important exclusions exist. - Copayments for public health services are low for category C and D if they make use of public health care. - Groups C and D may opt to use private providers within FONASA agreement however the co-payments are much higher. - FONASA allocates health services using quantity rationing (lines and waiting lists) rather than price rationing | - Per capita | - Care provided by public institutions fully covered by insurance for A and B category; Category C 10% & D 15% copayment - C and tier D using private sector: 50% co-payment |
|  | Private Health Insurance | ISAPRES (Instituciones de Salud Previsional), Armed Forces and Teachers Union | 1981 | Workers whereby their financial contributions match their health risk; in 2005, coverage was approximately 19% | - Main source of contributions (premiums) paid by members (93% between 1990 and 1997) equating to at least 7% of salary. Premiums are adjusted to match health risk to contribution, and beneficiaries are also able to purchase additional cover so premiums frequently exceed 7%. - 4% made up of sale of voluntary plans sold supplementary to plans those with compulsory contributions. - Premiums 7% but adjusted to match health risk to contribution - reviewed annually - Equity concern because insurers select young and healthy people; ISAPREs are able to decide whether to accept an individual as a beneficiary after gathering information about the person. | - All services provided under FONASA plus additional services that ae chosen by the beneficiaries based on plans purchased. - Since 2005, both private and public insurers were legally required to provide a similar benefit package covering certain legally defined health programs (including Schizophrenia; Depression in individuals over 15 years; Drug and alcohol dependence in adolescents from 10 to 19 years. | Not reported | - Insurance plans among individuals in the private cover- age group involved restrictions and variable copayments depending on the premiums paid. |
| Thailand | CBHI | Health Card Scheme | 1983 | Near poor and middle-income classes in rural areas; in 2000, coverage was approximately 30% of the Thai population | - Voluntary Health insurance card: Baht 1,000 or $40 per year per household of not more than 5 members [2]. - Household contributes half and other half subsidized by general tax revenue through the Ministry of Public Health - Specific time for card sales: cycle is one year and sale depends on seasonal fluctuations in income. - Premium is collected when cash incomes are highest (e.g when crops are harvested) - This scheme covered the near-poor population group on a voluntary basis, so there were some problems of selection bias [1, 3]. - Amalgamated into UHC scheme in 2002 | - Beneficiaries must register and seek care at certain first-contact health facilities, either a health center or a hospital. Anyone who bypasses this system must pay out of pocket [1][2]. | - Per capita | - No copayment provided referral procedures are followed and public facilities are used. |
|  | NHI | Universal Health Coverage scheme (previously 30 Baht Scheme) | 2002 | The rest of the population not covered by SSS and CSMBS; coverage 76.6% of the Thai population. The UHC Scheme replaced the Health Card Scheme in 2002. | - Entirely funded by government, mostly through general tax revenue. - Other contributions include those from local governments, fines from violating the UHC act, donations and interest on assets [1, 3]. | - Comprehensive package with 15 conditions excluded. Excluded conditions are: Psychosis except acute attacks; Drug addiction; Long-term hospitalization (more than 180 days in a year) - Choice of provider limited and must register with first-line provider in vicinity of residence or workplace - No copayments unless non-emergency services used from non-registered facilities. | - Capitation + Diagnosis-Related Group (DRG) | - 30-baht co-payment abolished in 2006 - No copayment at registered hospitals (includes prescribed medicines) provided referral procedures. |
|  | SHI | Social Security Scheme (SSS) | 1992 | Private sector employees (13% of the Thai population) | - Financed by equal contribution from employees, employers and the government - The scheme collects contributions from employees and employers equivalent to 1.5% of the salary of the employee. The government matches the contribution with 1.5% and resources are pooled at the national level [1, 3]. | - Comprehensive package with nonwork related illnesses; 15 conditions excluded. Excluded conditions are: Psychosis except acute attacks; Drug addiction; Long-term hospitalization (more than 180 days in a year) - Moderate limitation in choice of provider, registration required with first-line providers, but with more choices. - Co-payments exist for maternity and emergency services if beyond a budget ceiling. - Includes medical care and cash benefits: 50% of wages 90 days at a time up to a maximum of 180 days/ for chronic cases | - Contract capitation system; use Diagnosis-Related Group in risk adjusted part | - No copayment at registered hospitals (includes prescribed medicines) provided referral procedures are followed |
|  | SHI | Civil Service Medical Beneﬁts Scheme (CSMBS) | 1960s | Govt employees & dependents, retirees (7% of Thai population) | - Tax-revenue financed, resources are centrally pooled at the National level. - CSMCS is a government fringe benefit package financed by taxes | - Comprehensive package; no conditions excluded. - Almost unlimited provider choice and can go to - any public facility. - Co-payments exist for inpatient care at private hospitals. | - Fee-for-service for Outpatient, and Diagnosis-Related Group for Inpatient | - No copayment at registered hospitals (includes prescribed medicines) provided referral procedures are followed |
| South Korea | NHI | Korean national health insurance | 1977 (full geographical coverage by 1989) | The population of Korea; 96.4% of the population is covered by the NHI | - Combination of NHI contributions and government taxes - Government subsidies to NHI at prescribed level of 20% of NHI revenues from contributions - NHI covers about 60% of medical expenditure incurred by their beneficiaries. - Two categories of NHI insures: (1) the employees (industrial workers, government employees and teachers): 5.08% of salaries for the EE insurees; with employer and employee each paying half; (2) self-employed (daily workers who are employed less than one month a year, military personnel and elected public officials without a monthly salary, or part time workers) - Household's total income is assessed, income types assessed at different weights: ₩50,513 per household or ₩21,594 per insured person; ₩2,000 minimum contribution - In 2000, all insurance schemes merged into single payer with uniform contribution schedule and benefits package [4] | - NHI benefits are provided for the prevention and treatment of disease and injury, for childbirth, and for health promotion and rehabilitation, but the focus is on curative care | - By law, all hospitals and clinics, whether public or private, as well as pharmacies, are obliged to subscribe as providers under the NHI and cannot opt out. - Fee-for-service rate fixed regardless of services psychiatric inpatients receive - DRGs are applied only to a limited number of diseases and the participation of facilities is voluntary. - Patients pay special treatment charges for being treated by doctors with a certain amount of work experience and special room charges for admissions to a better-equipped hospital room with fewer than five beds. This applies to psychotherapy where patients pay up to 50%; or 100% for long terms therapy. | - A 20% co-payment is required for inpatient care services included in the benefit package, but this ranges from 30% to 60% for outpatient care, depending on the level of provider - The poor are exempted from cost-sharing at the point of service, and vulnerable patient groups have access to discounted copayment rates. - Full payment for services not included in the benefits package |
|  | Government subsidies for those who do not have economic capability, and cannot work | Medical Care Aid 1 | 1977 | Those who do not have economic capability; the beneficiaries who are not capable of working are categorized as AID Type 1 (2.1% population coverage). | - Government covers 100% of medical expenditure incurred by AID Type 1 beneficiaries. - Medical Aid Program financed by general revenue of the central and local governments but administered (including payments to providers) through health insurance system. - Previously exempted both from paying contributions and cost-sharing. Since 2007, specific cost-sharing schedule in place for them. [4] | ?? | - For psychiatric inpatient care institutions are reimbursed for AID beneficiaries at per-diem rates |  |
|  | Government subsidies for those who do not have economic capability, and can work | Medical Care Aid 2 | 1977 | Those who do not have economic capability; the beneficiaries who can work are AID Type 2 (1.6% population coverage) | - Government covers 85% of medical expenditure incurred by AID Type 2 beneficiaries (1.6% of population) - Cost-sharing higher than for type 1 members but lower than for regular NHI beneficiaries [4] | ?? | - For psychiatric inpatient care institutions are reimbursed for AID beneficiaries at per-diem rates |  |
|  | Government subsidies for Veterans | Veterans Health | 1977 | Veterans, coverage rate not reported | Not reported |  |  |  |
| China | SHI | Urban Employee Basic Medical Insurance (UE-BMI) | 1998 | UE-BMI targets formal sector workers on a mandatory basis; coverage for UE-BMI is 19% of the population. | - UEBMI mandatory and administrated at municipal level - Funds of UEBMI came from 8% of the employee's wage: 6% by employers and 2% by employee (rates vary by time and municipalities) | - UEBMI covers both outpatient and inpatient health services; no copayment. - Health care and drug packages covered by UEBMI more generous than the other schemes - UEBMI drug and health care package developed and implemented by the municipal cities which are the unit of fund pooling. - Approximately 2000 drugs covered by the Urban Schemes | - UE-BMI moved from patient reimbursement to capitation payment to designated providers for outpatient services - Providers paid standard 80 RMB per person per month from the risk-pooling fund for providing outpatient services. - UEBMI still reimburses members on a fee-for-service basis for acute inpatient admissions. - For long-term inpatients with specified serious conditions, it pays “fee for unit according to hospital level” 120 RMB/day (tertiary), 110 RMB/day(secondary) and 70 RMB/day (primary). | - No copayment |
|  | SHI | Urban Residence Basic Medical Insurance (UR-BMI) | 2007 | UR-BMI targets children, the elderly, the disabled, and other non-working urban residents but varies by region. Enrollment is voluntary for households; coverage for UR-BMI is 19.5% of the population* | - URBMI is government-subsidized, household-level-voluntary medical insurance, administrated at municipal level. - Funds of URBMI mainly from individual contributions (245 yuan for adults), and government contributions (at least 80 yuan per capita) [5]. - Additional government contributions given to undeveloped central and western regions and poor or disabled individuals | - URBMI covers inpatient care only; 35-55% copayment. - Approximately 2000 drugs covered by the Urban Schemes | - Fee-for-service | - 35-55% copayment |
|  | SHI | New Rural Cooperative Medical scheme (NCMS) | 2002 | NCMS targets rural residents on a voluntary basis; coverage for NCMS is approximately 59.7% of the population * | - NCMS is a voluntary insurance scheme subsidized by the local and central government. - Administration and risk-pooling set at county level - Funds of NCMS provided by local and central government (for poorer regions) together | - NCMS covers both outpatient and inpatient care in about 70% of the NCMS counties, the other 30% offering coverage for inpatient care only. - Design and implementation of health care package and drug lists is mainly the responsibility of each of the NCMS counties that are the unit of fund pooling and management. - 400 drugs covered by the NCMS | - Fee-for-service | - NCMS covers expenses in all public healthcare facility levels (rate varies by regions and by type of facilities). |
|  | GHI | Government Insurance System | <1980 | Not reported | Not reported | Not reported | Not reported | - GIS provides almost complete payments, very limited copayments. |
| 22 low-income,  17 lower-middle, and  9 upper-middle countries (2003) | SHI or NHI | Countries where most or all health services, including primary care, are provided by the government (even if private or NGO sector services may exist in parallel and some out-of-pocket expenses may exist). | N/A | N/A | N/A | N/A | N/A | N/A |
|  | Private health insurance | Countries with no or minimal services provided by the government, or where only limited health services were provided by the government (e.g., for maternal and child health, HIV/ AIDS care, vaccinations, or for special groups such as children, elderly, impoverished). | N/A | N/A | N/A | N/A | N/A | N/A |
| *In 2016, the urban resident and rural schemes merged to form the urban rural resident basic medical insurance (URRBMI) to improve administrative efficiency[6] | | | | | | | | |

1. Gottret P, Schieber G, Waters HR. Good practices in health financing: lessons from reforms in low and middle-income countries: The World Bank; 2008.

2. Pannarunothai S, Srithamrongsawat S, Kongpan M, Thumvanna P. Financing reforms for the Thai health card scheme. Health Policy and Planning. 2000;15(3):303-11.

3. Boon-Arj C. Universal Coverage in Access to Health Care: National experiences of Thailand N.d [cited 2019 21 September]. Available from: <https://www.social-protection.org/gimi/RessourceDownload.action?ressource.ressourceId=13112>.

4. Mathauer I, Xu K, Carrin G, Evans DB. An analysis of the health financing system of the Republic of Korea and options to strengthen health financing performance. World Health Organizaiton Geneva: Swiss. 2009:29-30.

5. Health Insurance in China OnlineN.d [cited 2019 21 September]. Available from: <https://en.wikipedia.org/wiki/Health_insurance_in_China>.

6. Fang H, Eggleston K, Hanson K, Wu M. Enhancing financial protection under China’s social health insurance to achieve universal health coverage. BMJ. 2019;365:l2378. doi: 10.1136/bmj.l2378.
