# Supplementary figures and images for: The impact of social, national and community-based health insurance on health care utilization for mental, neurological and substance-use disorders in low- and middle-income countries: a systematic review
Source: Health Econ Rev. 2020 Apr 24;10:11. doi: 10.1186/s13561-020-00268-x (PMC7181535; doi:10.1186/s13561-020-00268-x)

Additional File 3: Graphical Visualization of Key Findings

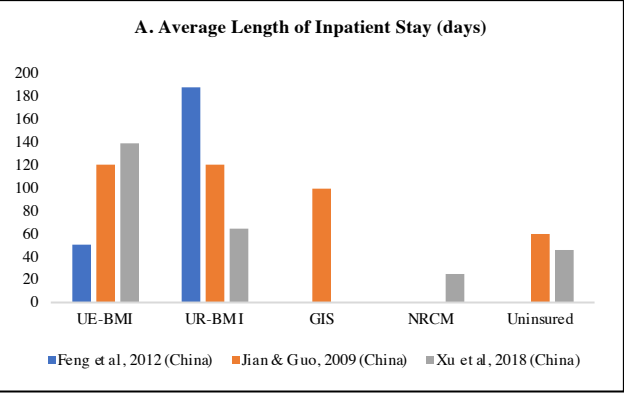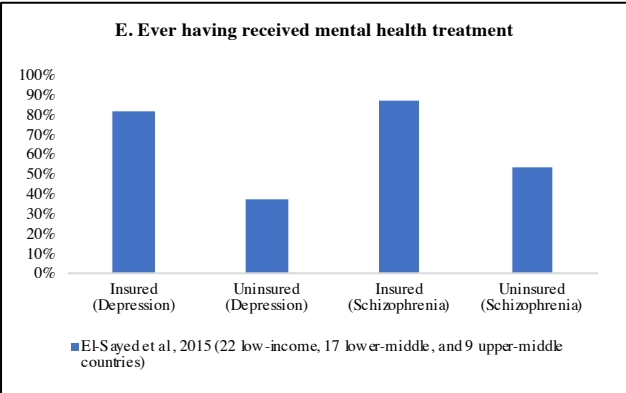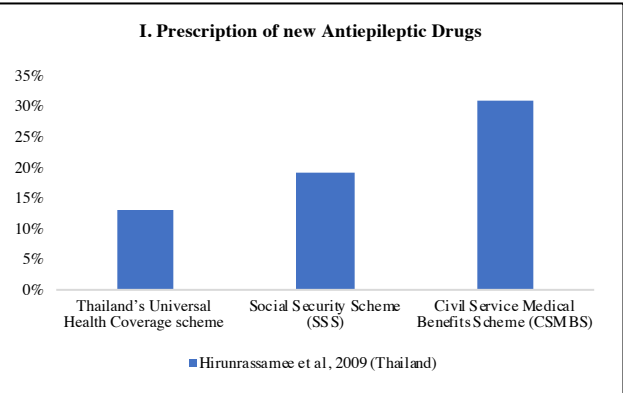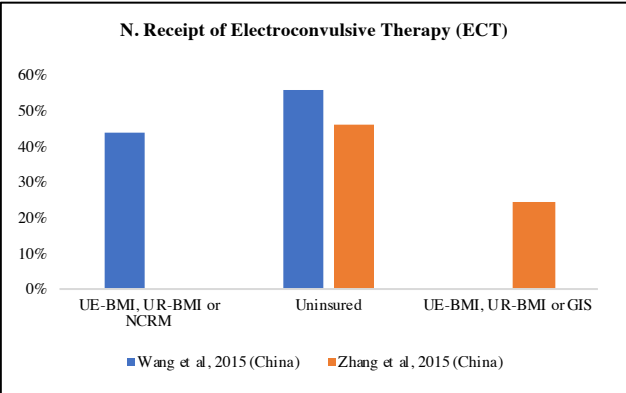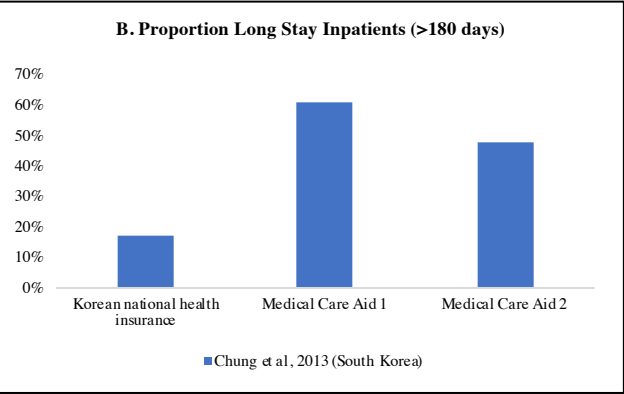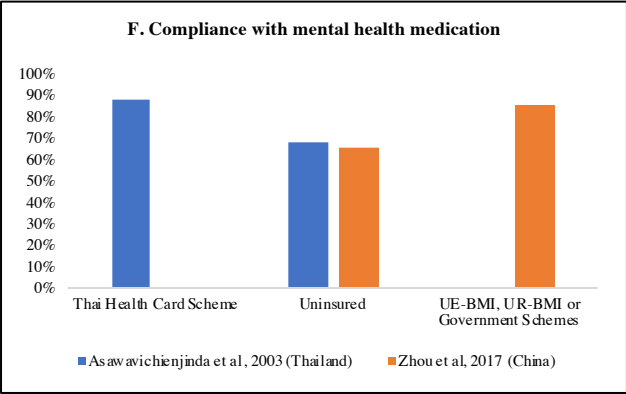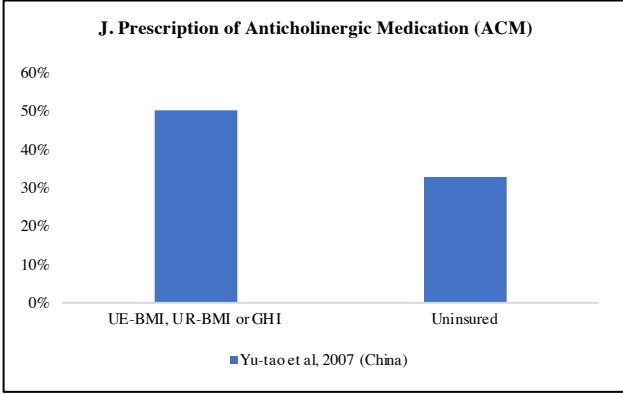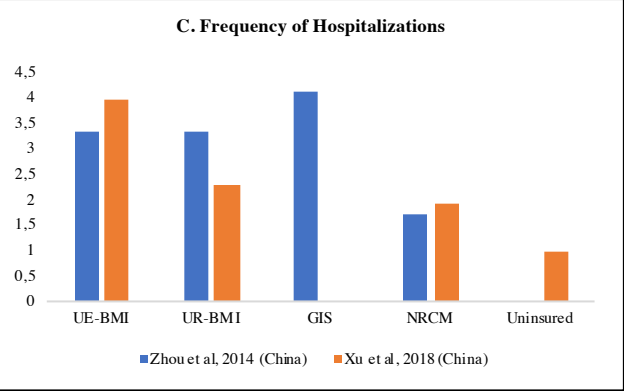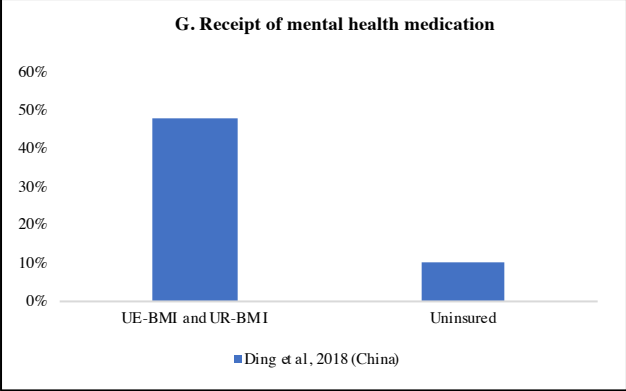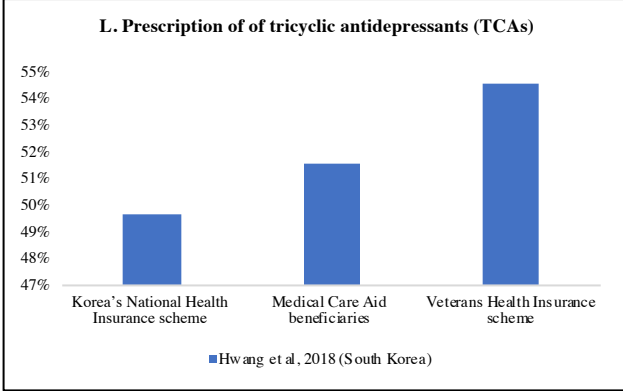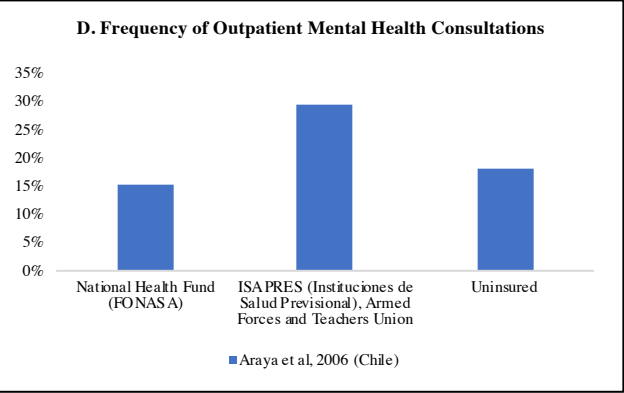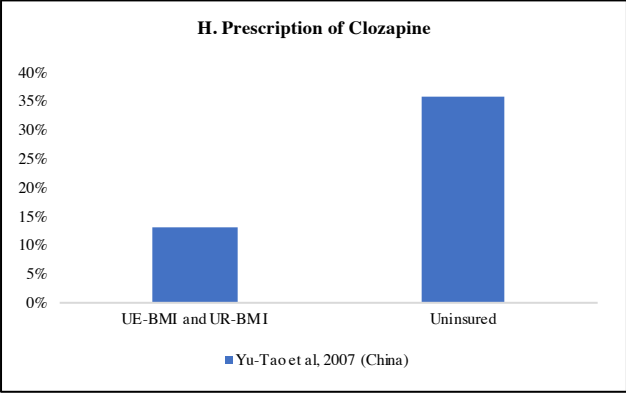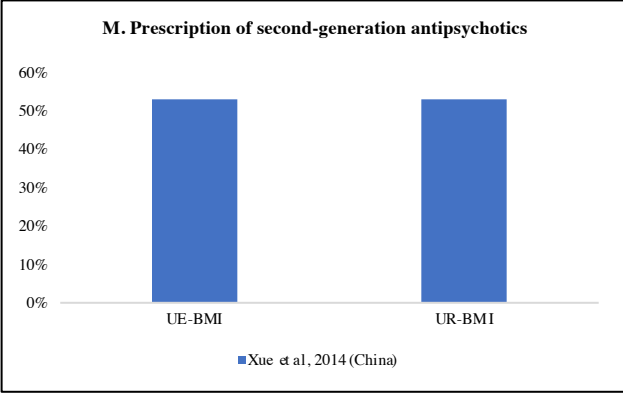

Supplement: Supplementary file 3 — Additional file 3. [file 13561_2020_268_MOESM3_ESM.pdf]
